# Procedural Optimisation and Unmeasured Implementation in External Cephalic Version at Term: A Narrative Review of a Measurement Asymmetry

**DOI:** 10.3390/healthcare14203452

**Published:** 2026-10-15

**Authors:** Alexandru Cărăuleanu, Vasile Adrian Sescu, Claudia Florida Costea, Raluca Haba, Iustina Petra Solomon Condriuc, Raluca Anamaria Mogoș, Răzvan Socolov, Demetra Socolov

**Affiliations:** 1 Grigore T. Popa University of Medicine and Pharmacy, 700115 Iasi, Romania; ale.carauleanu@umfiasi.ro (A.C.); raluca.haba@umfiasi.ro (R.H.); razvan.socolov@umfiasi.ro (R.S.); demetra.socolov@umfiasi.ro (D.S.); 2 Department of Obstetrics and Gynecology, Cuza Voda Hospital, 700038 Iasi, Romania; 3 Department of Ophthalmology, Emergency Clinical Hospital “Prof. Dr. Nicolae Oblu”, 700309 Iasi, Romania; 4 Department of Obstetrics and Gynecology, Clinical Hospital of Obstetrics and Gynecology “Elena Doamna”, 700038 Iasi, Romania

**Keywords:** health services research, implementation science, process indicators, clinical audit, maternity care, caesarean section, external cephalic version, breech presentation

## Abstract

Caesarean delivery is rising worldwide, and for breech presentation at term, external cephalic version (ECV) reduces non-cephalic presentation at birth and, at very low certainty, caesarean delivery. Procedural evidence is reported per attempt, whereas losses determining whether an eligible woman reaches an attempt occur earlier and, in the literature we retrieved, are rarely measured. This narrative review compares procedural evidence with what is recorded about progression to ECV. Immediate success occurs in roughly half to six in ten attempts. Severe procedure-attributable complications are uncommon under strict definitions, at 0.24% in pooled data and 0.45% in a large series, whereas broader short-term composites reach 6–10% and count different events. Attempt rates were 62.2% and 72.5% of eligible women in two Dutch cohorts and 7.9% of a source-defined cohort in United States data, over non-comparable denominators. No contemporary study identified in our search reports the proportion of eligible women offered ECV in ordinary clinical practice, with eligibility assessed in every term breech presentation, despite an auditable standard targeting 100%. We identified no study measuring procedural effects and losses before the attempt within one population. Utilisation varies markedly, but the extent of any underuse cannot be quantified from this documented, non-exhaustive search. We set out an author-derived nine-state pathway with explicit denominators and three proposed process indicators as a reporting framework, not as validated measures.

## 1. Introduction

Caesarean delivery is now performed in more than one in five births worldwide, with nationally representative data from 154 countries placing the rate at 21.1% with a projected 28.5% by 2030 [1]. A first caesarean creates a uterine scar whose risks accrue across a woman’s reproductive career [2], and breech presentation is a quantitatively meaningful entry point into that sequence: in a systematic review of European practice using the Robson ten-group classification, between 88.8% and 92.5% of nulliparous women with a singleton breech pregnancy were delivered abdominally [3].

Breech presentation persists at term in about 2.5–3% of singleton pregnancies [4]. External cephalic version (ECV) is the principal antenatal intervention for this group. The Cochrane review of randomised trials and a systematic review restricted to high-resource settings both find that attempting it reduces non-cephalic presentation at birth and caesarean delivery, the latter at very low certainty; Section 4 sets out the estimates [5,6]. The major professional societies whose current documents address the procedure recommend that it be offered to eligible women at term [7,8,9,10].

The technical evidence base is comparatively mature: predictors of success have been catalogued and modelled [11], adjuncts tested in randomised trials, and an integrative synthesis concluded that ECV is safe and effective and called for wider adoption [12], a conclusion resting, as Section 4 sets out, on strictly defined complication outcomes. Utilisation is less well documented and appears to vary widely. ACOG states in its own practice bulletin that an estimated 20–30% of eligible women are not offered the procedure [7], and United States national data record an attempt in 7.9% of a source-defined cohort [13]. In the Netherlands, a system with an explicit national guideline and an organised referral structure, the attempt rate among eligible women ranged from 8% to 95% between clusters [14,15]. These figures are expressed over different denominators and should not be read as directly comparable measures of service performance. Section 10.2 sets out these figures and the denominators they rest on. Barriers at the level of the woman, the clinician and the service have been characterised in dedicated work [16,17].

These two bodies of evidence are not expressed over the same denominator. That difference is the organising problem of this review. Procedural estimates are conditional on an attempt having been made: they describe what happens to the women who receive the procedure. Implementation quantities begin one step earlier, with the eligible population, and describe how many women receive it at all. Because the second denominator is rarely recorded in the studies we retrieved, the procedural literature operates inside a population whose size is fixed by decisions it does not measure. We refer to this imbalance as a measurement asymmetry; it is the organising framework of this review. On the implementation side, a key quantity is the offer rate, the proportion of eligible women offered ECV when eligibility has been assessed in every woman with a term breech presentation (an eligibility-assessed denominator). Ordinary clinical practice denotes routine care, as distinct from study protocols that offer ECV to every eligible woman; Section 10.1 defines the pathway and gives the operational definitions.

This narrative review has two objectives: to synthesise current evidence on the determinants of ECV success and on the interventions that improve it, and to set that evidence against what is recorded about how often eligible women are identified, counselled, offered and given the procedure. The scope is restricted to singleton pregnancies at term with breech presentation or transverse lie. ECV before term is excluded, with the relevant trials addressing a different risk–benefit trade-off that includes an increased risk of late preterm birth [18], as is ECV of the non-cephalic second twin, an intrapartum procedure performed under different conditions [19]. Intrapartum ECV, ECV with ruptured membranes and patient-reported outcomes other than pain are not covered, as too little evidence has been identified to support a useful synthesis.

The procedural evidence is set out at some length, and the reason is not clinical completeness. A service cannot be said to be underusing an intervention until it is known what that intervention delivers when it is used well, and at what cost in harm. Section 4, Section 5, Section 6, Section 7, Section 8 and Section 9 therefore establish the benchmark: how often the procedure succeeds, what raises that probability, what it costs the woman in risk, and what the professional bodies commit their members to. Section 10, Section 11 and Section 12 set the organisational evidence against that benchmark, and the denominator over which each quantity is expressed is stated wherever the two are placed side by side.

## 2. Materials and Methods

This narrative review was prepared with reference to the SANRA criteria [20]. PubMed/MEDLINE, Scopus, Web of Science and the Cochrane Library were searched from inception to 20 August 2026, combining terms for external cephalic version with terms for breech presentation and transverse lie, and separately with terms for implementation, utilisation, uptake, counselling and health-services access. Systematic reviews, meta-analyses, randomised trials and current guidelines were given priority for efficacy, safety and procedural questions, these being questions about the effect of an intervention, for which controlled designs and pooled syntheses give the least biassed estimates. Implementation questions required a broader evidence base, because pathway measures such as identification, counselling, offer and attempt rates are frequently reported in service-level and population data rather than in controlled trials. Observational studies were retained where they supplied implementation or denominator data unavailable from higher-level designs, and where they reported at least one step of the pathway defined in Section 10.1 over an identifiable denominator, which was the operative inclusion criterion for an implementation study. Design was not itself a criterion, so that population cohorts, service audits, before-and-after evaluations, clinician surveys and qualitative studies are all represented; their differing evidential weight is recorded for each entry in the Supplementary Materials and is not to be read as equivalent. Evidence falling partly outside the term scope set out in Section 1 was retained only where it bore directly on the implementation question. It is presented as contextual where it is used, as for the late-preterm cohort in Section 10.7, and is not relied on for conclusions about term practice. Reference lists were screened and forward citation searching used for the principal implementation studies. Consistent with the narrative design, no study flow diagram or record count is reported.

For the guideline comparison, the unit of tabulation was a graded recommendation on external cephalic version. Supplementary Tables S1 and S2 set out the four documents that recommend across the domains compared. Two further national documents govern term breech care without doing so, and Section 8.4 treats them separately. The division is substantive and not practical. A document may be current, authoritative and squarely relevant to term breech management while saying little or nothing about the procedure, and tabulating it yields a row of blanks that misrepresents what it is. Documents were sought from the major professional bodies whose guidance circulates internationally, and from the health systems contributing utilisation data to Supplementary Table S3. Content was taken from the issuing documents, including their published summary statements, and not from secondary comparisons. The comparison is illustrative and not an exhaustive survey of national guidance. For the Romanian guideline, the full text and reference list were checked for the procedure under both its Romanian and its English names before the absence was recorded.

As a narrative review, this article involved no protocol registration, no formal risk-of-bias assessment, no certainty-of-evidence grading by the authors and no quantitative pooling; certainty ratings reproduced in the text and tables are those assigned by the source review. Article selection and data extraction were performed by a single reviewer, without duplicate screening, and the search, although documented, was not exhaustive. Statements in this review that a quantity was not reported therefore describe the literature retrieved and are not claims that no such report exists. Effect estimates supporting the procedural sections are tabulated rather than repeated in the text; Table 1 and Table 2 and Supplementary Tables S1–S4 should be read alongside Section 5, Section 6, Section 7, Section 8, Section 9 and Section 10.

## 3. Term Breech Presentation: Clinical Context

Breech presentation is common in the second trimester and uncommon at term: in a Finnish national cohort of 737,788 singleton births, incidence fell from 23.5% at 24–27 weeks to 2.5% at term. Most foetuses undergo spontaneous version, and the residual term population is small, consequential and selected, since advanced maternal age, nulliparity and previous caesarean all favour persistence to term [4].

The management of this group changed abruptly in 2000. The Term Breech Trial reported lower perinatal mortality and serious neonatal morbidity with planned caesarean [36], and practice followed within months. The Dutch caesarean rate for term breech rose from 50% to 80% within two months [37], and a national cohort of 58,320 deliveries documented elective caesarean rising from 24% to 60% with a fall in perinatal mortality, at 338 caesareans per perinatal death prevented [38]. The two-year follow-up complicated the message, with planned caesarean conferring no advantage in death or neurodevelopmental delay [39], and the PREMODA survey of 8105 women found no significant difference where selection and intrapartum criteria were applied strictly [40]. Contemporary meta-analytical data nonetheless favour planned caesarean for the neonate, at the cost of greater severe maternal morbidity [41]. Where planned vaginal breech birth is unavailable, caesarean is the practical counterfactual for every woman whose foetus remains breech.

The decision also extends beyond the index pregnancy. In a Danish register-based cohort of 30,778 nulliparous women with a term breech singleton, planned caesarean was associated with lower odds of postoperative infection, but with higher odds of uterine rupture in the second pregnancy (adjusted odds ratio [aOR] 1.85; 95% CI 1.37–2.50) and the third (aOR 3.15; 95% CI 1.08–9.24) [42]. Across all indications, a pregnancy following caesarean carries increased odds of placenta praevia, placenta accreta and placental abruption [2]. A successful ECV may avert a primary caesarean and, with it, exposure to scar-related risk in subsequent pregnancies. It does not guarantee vaginal birth, and the benefit is cumulative across a reproductive career, which is the grounds on which the organisational effort the procedure demands is usually justified.

## 4. Efficacy, Safety and Cost-Effectiveness

A Cochrane review of eight randomised trials (1308 women) found that attempting ECV reduced non-cephalic presentation at birth (average RR 0.42; 95% CI 0.29–0.61), failure to achieve a vaginal cephalic birth (average RR 0.46; 95% CI 0.33–0.62) and caesarean delivery (average RR 0.57; 95% CI 0.40–0.82). Certainty was graded very low for the latter two outcomes, the trials being small and of variable quality rather than discordant in direction [5]. A systematic review covering 184,704 breech pregnancies in high-resource settings produced a closely comparable caesarean estimate (RR 0.57; 95% CI 0.50–0.64) [6].

What varies is the probability of technical success: a meta-analysis of 84 studies reporting 12,955 attempts gave a pooled success rate of 58%, with individual estimates from 16% to 100% and substantial heterogeneity (I^2^ = 94%) [43], and contemporary cohorts span 22.4% to 71.7% (Section 10). Section 5, Section 6 and Section 7 address the determinants of that dispersion.

Immediate success is not the outcome that matters clinically. In a United Kingdom cohort of 2614 women undergoing ECV, 97.3% of successfully verted foetuses were cephalic at birth and 85.7% of those delivered vaginally, with reversion occurring in 2.2% [44]; combining the two proportions, approximately four in five achieved a vaginal cephalic birth. That figure combines proportions reported over slightly different denominators and is an approximation; a Spanish cohort reported 80.5% directly [22]. The compound probability, not the immediate success rate, belongs in counselling.

Safety must be reported in absolute terms with the outcome definition stated, because published rates differ by more than an order of magnitude according to how the outcome is defined. Two tiers should be kept apart. Across 12,955 attempts, the pooled meta-analysis reported serious complications in 0.24% (95% CI 0.17–0.34) and procedure-attributable emergency caesarean in 0.35% (95% CI 0.26–0.47), with no association between complications and version outcome [43]. An Australian series of 1121 procedures applied a published event list, comprising foetal death, placental abruption, foetal distress requiring emergency caesarean, foetal bone injury and cord prolapse, and recorded five such events (0.45%), one a foetal death after a successful version [45]. One pooled and one explicitly defined estimate therefore agree at well under 1%.

Broader short-term composites give higher figures. The same meta-analysis reported any complication in 6.1% (95% CI 4.7–7.8) [43] and the Australian series reported a further rate of 4.28% of minor events [45]. A Spanish cohort of 990 procedures reported complications in 10.2% [22], and a Canadian tertiary centre attributed emergency caesarean to the procedure in 10% of 258 procedures [28].

These contemporary figures are not anomalies: three services in three countries agree. Nor are they comparable with the pooled 0.24% and 0.35%, counting different events over different windows, some extending to birth. The pooled estimates themselves aggregate 84 studies retrieved to 2007, each applying its own definition [43]. The position taken here is narrow: severe procedure-attributable complications are uncommon under strict definitions.

The studies pooled in the risk meta-analysis were heterogeneous and largely observational, and are now approaching two decades old. The high-resource systematic review reported a small increase in Apgar score below 7 at five minutes (RR 1.29; 95% CI 1.10–1.52) [6], which belongs in counselling.

At population scale the downstream contrast is large. Across 32,321 singleton breech pregnancies in New South Wales, caesarean delivery followed 25.9% of successful and 95.6% of unsuccessful attempts, with no difference in infant outcomes by success [46].

The economic case is favourable but not unconditional. A decision-analytic model adopting a societal perspective found a trial of ECV to be cost-effective relative to scheduled caesarean provided the probability of success exceeded 32% [47]. Most success rates reviewed here lie above that threshold; two do not, at 31% [28] and 22.4% [48], and a service at those rates is at or below the modelled break-even. The model used 2007 United States costs and a $50,000 threshold per quality-adjusted life-year (QALY), so its absolute figures do not transfer, although its structural conclusion does; the only subsequent evaluation identified outside the United States reached the same qualitative conclusion [49].

## 5. Determinants of Procedural Success

Predictors of a successful ECV have been catalogued repeatedly and the models built from them synthesised systematically [11]. Two meta-analyses provide the quantitative backbone, pooling clinical prognosticators from 53 studies of 10,149 women [21] and ultrasound prognosticators from 37 studies of 7709 women [24]; effect estimates are set out in Table 1 and are not repeated here except where the argument turns on their magnitude.

Parity is the most consistently reported determinant, with multiparity more than doubling the odds of success, with a smaller advantage for maternal weight below 65 kg [21]. Neither meta-analysis pooled body mass index, although contemporary cohorts report it as an independent predictor of failure [22,23]. Greater maternal weight reduces the probability of success, but the evidence defines no threshold above which an attempt should be withheld.

The ultrasound determinants are real but comparatively weak, each raising the odds of success by a factor below two and a half [24]. The largest pooled associations in that literature are obtained by hand: a relaxed uterus, non-engagement of the breech and a palpable foetal head, each with a wider effect than any sonographic parameter (Table 1) [21]. Clinical examination at the attempt therefore carries more prognostic weight, and a sonographic measure of engagement has not matched it: in 117 women the transperineal breech progression angle was associated with success but discriminated poorly [27].

Neither gestational age at the attempt nor fundal height was associated with the outcome [21]. Consistency across settings is imperfect: in a cross-sectional series of 152 procedures in Ethiopia, where success was 71.7%, neither parity nor engagement reached significance [26].

Two pooled determinants have been contradicted by individual cohorts: a Dutch cohort of 673 women found complete breech associated with lower success, opposite to the pooled ultrasound estimate [25], and a Canadian review found uterine relaxant use associated with lower success, attributed by its authors to confounding by indication [28]. Single-centre analyses of this kind do not carry the weight of pooled estimates.

Prediction models built on these determinants have largely not translated into practice. Of 25 models identified in an updated systematic review, only the Newman–Peacock model, externally validated more than once, was judged ready for regular clinical use [11]. Table 1 is therefore set out as individual associations and not as a model.

## 6. Procedural Optimisation: Timing, Technique and Case Selection

### 6.1. Gestational Age at the Attempt

Because gestational age does not predict technical success [21], the timing decision rests on a trade-off between presentation at birth, reversion and the risk of iatrogenic preterm birth. The Early External Cephalic Version 2 Trial randomised 1543 women to ECV at 34–35 weeks or at 37 weeks or later: fewer foetuses were non-cephalic at birth in the early group (RR 0.84; 95% CI 0.75–0.94) and the caesarean rate did not differ significantly [50]. Pooling that trial with two others, the Cochrane review reproduced the presentation benefit, again found no significant caesarean reduction, and found a significant increase in late preterm birth (RR 1.51; 95% CI 1.03–2.21), at high certainty [18].

This is the evidential basis for the term-onwards default that most guidance adopts. Thresholds differ between documents, from 36 weeks to 37 + 0 weeks (Supplementary Table S1) [7,8,9,51,52]. The trade-off should be stated to women in these terms and not presented as an institutional convention.

### 6.2. Technique, Ultrasound and Monitoring

The manoeuvre is the least standardised element of the procedure, and the Cochrane review names its standardisation as a research priority [30]. No randomised comparison of the forward roll with the back flip was identified; ACOG describes the forward roll [7]. Two guidelines advise no more than four attempts and a maximum of ten minutes overall, both noting that the evidence guiding this is limited [8,9]. The manipulation is agreed to be gentle and sustained, with disengagement of the breech before rotation, and is abandoned on maternal request or for a non-reassuring foetal heart rate.

Significant pain was reported by 34% of women in a Dutch primary-care cohort, and 18% of those also reported fear, against none of those reporting little or no pain [53]. The absence of pain and a soft uterine tone were both associated with success [26], consistent with the pooled finding for a relaxed uterus [21]. Maternal pain, uterine tone and technical failure are plausibly three observations of one phenomenon, the mechanistic argument for the tocolysis and analgesia Section 7 quantifies.

Pre-procedural ultrasound is indispensable for reasons that are not prognostic: it confirms the presentation and its type, documents placental location and amniotic fluid volume, estimates foetal weight and excludes anomalies [7]. Cardiotocography before and after the procedure, and facilities for monitoring and surgical delivery, are specified in the guidance (Supplementary Table S2) [7,51,52]; the justification is the low but non-zero rate of procedure-attributable emergency caesarean [43], not trial evidence that monitoring alters outcome. Standard preoperative preparations for caesarean, routine fasting, anaesthetic premedication and intravenous access other than for tocolysis are explicitly not recommended [8,9]. Treating ECV as a quasi-surgical event may increase resource requirements and restrict access without established evidence of additional safety benefit.

### 6.3. Anti-D Prophylaxis

ECV is a potentially sensitising event, and non-sensitised RhD-negative women should receive anti-D immunoglobulin afterwards; the British guideline specifies a minimum of 500 IU within 72 h with a test for fetomaternal haemorrhage [54]. RANZCOG and ACOG both specify anti-D whether or not the ECV succeeded [7,9]; doses differ between jurisdictions and the national document governs [55]. Systematic Kleihauer–Betke testing after 282 attempts was positive in 1.8%, with no adverse outcome, and was judged probably not useful [56]. The CNGOF has adopted that position while requiring that prophylaxis be assured [51,52].

### 6.4. ECV After Previous Caesarean Delivery

A uterine scar has long been treated as a relative contraindication, but a systematic review of women with at least one previous caesarean found ECV successful and low in risk, with no included study reporting uterine rupture [57]. In United States birth-certificate data, success after one previous caesarean was 80.6% against 86.4% in unscarred women; transfusion and unplanned hysterectomy were more frequent among those who then attempted labour [58], with the risks attaching to the trial of labour rather than to the ECV. In a Spanish cohort of 911 procedures, success after previous caesarean was 78.6%, with no difference from the unscarred group after adjustment [59]. The evidence is observational and collectively underpowered for an event as rare as scar dehiscence, as the RCOG states explicitly [8]. A previous caesarean is not by itself a reason to withhold the offer, provided the woman is counselled that the evidence is weaker than for an unscarred uterus.

### 6.5. Operator Experience and Service Organisation

The determinants reviewed in Section 5 are properties of the woman; those that follow are properties of the service. A learning curve is consistently described, but its length is disputed. An early series of 80 procedures reported a plateau after the first 20 cases [60], whereas a cumulative-sum analysis of 290 procedures found that 8 to 10 sufficed for competence in multiparous women but 57 were needed for a consistent 50% success rate in nulliparous women and 130 for 70% [29]. The two used different methods and definitions of competence and neither has been replicated in the literature retrieved, so together they are consistent with a substantial volume requirement in nulliparous women without establishing a threshold. A Canadian review found no association between provider seniority and success [28], whereas a Dutch hospital that reorganised ECV into a dedicated team recorded a rise in success, the largest gain in nulliparous women [25]; both are uncontrolled. Seniority and volume are not the same variable, and only the second is modifiable by organisation. Section 11 sets out the figures and the wider organisational evidence.

## 7. Pharmacological and Anaesthetic Adjuncts

Table 2 sets out the randomised evidence for every adjunct considered below, with comparators, effect estimates, absolute effects and the certainty ratings assigned by the source reviews. The text states what follows clinically and repeats an estimate only where the argument turns on its magnitude.

### 7.1. Tocolysis

Parenteral beta stimulants are the only tocolytic class with consistent randomised support, and the effect extends beyond the procedure: in the Cochrane review they increased cephalic presentation in labour and reduced both caesarean delivery and failure to achieve a cephalic vaginal birth, the latter two at moderate certainty [30]. In direct comparison, calcium channel blockers were inferior for both cephalic presentation at birth and caesarean delivery and nitric oxide donors performed no better; these are single small trials, and the review judged the evidence insufficient to recommend either class [30].

In a meta-analysis of four randomised trials including 1534 women, success was significantly lower with atosiban than with beta-agonists, while caesarean and vaginal delivery rates did not differ and drug-related side effects were markedly fewer (Table 2) [33]. The better-tolerated agent is the less effective one.

### 7.2. Neuraxial Techniques

Neuraxial analgesia has one of the most consistent effect estimates for increasing immediate success. In a meta-analysis of nine randomised trials including 934 women, it increased successful ECV. Cephalic presentation in labour and vaginal delivery also rose, pain was substantially lower, and emergency caesarean, transient bradycardia and abruption did not differ (Table 2) [31]. The absolute difference of approximately 15 percentage points corresponds to roughly seven women receiving neuraxial analgesia for one additional successful ECV.

A network meta-analysis of 19 trials comprising 2296 women ranked neuraxial anaesthesia with a beta-stimulant highest and neuraxial anaesthesia alone next, both above beta-stimulants alone, with the caveat that the trials differed in technique, dose and definition of success (Table 2) [32].

The three syntheses disagree about caesarean delivery. The Cochrane comparison of regional analgesia plus a tocolytic against a tocolytic alone found failed ECV reduced at moderate certainty but there was no significant difference in caesarean delivery, with the trials being underpowered for that outcome [30]. Two of the three therefore find no demonstrable caesarean benefit and one does, and they differ in comparators, analytical model and outcome definitions. Neuraxial analgesia reliably raises the probability of turning the foetus and reduces pain, while its effect on caesarean delivery is unsettled.

This is the point at which procedural optimisation and implementation come into conflict. Neuraxial analgesia converts an ambulatory bedside procedure into one requiring anaesthetic staffing, a delivery-suite bed and a longer stay, so that where capacity rather than technique is the binding constraint, the intervention with the best evidence for raising success may also reduce the number of women to whom it can be offered. This tension returns in Section 11.

### 7.3. Adjuncts with Insufficient Evidence

The Cochrane review found the data insufficient for regional analgesia without tocolysis, vibroacoustic stimulation, transabdominal amnioinfusion, systemic opioids, hypnosis and abdominal lubricants [30]. None should be described to women as established.

### 7.4. Methods Attempted Before ECV

Moxibustion and postural management are alternatives attempted before ECV rather than adjuncts to it. The Cochrane review of moxibustion, updated to 13 studies and 2181 women, concluded that it probably reduces non-cephalic presentation at birth relative to usual care and to sham, both at moderate certainty, while its effect on the need for ECV remained uncertain [34]. For postural management the evidence remains insufficient [35].

Guidance diverges more sharply here than anywhere else in this review. The RCOG’s Grade C statement that women may wish to consider moxibustion under a trained practitioner sits against RANZCOG’s advice that recent trials show no evidence of effectiveness and an explicit Grade B negative recommendation from the CNGOF [8,9,51,52]. Chronology accounts for part of the spread, with the RCOG and RANZCOG statements predating the 2023 Cochrane update.

## 8. Guideline Recommendations, Eligibility and Contraindications

### 8.1. The Recommendation and Its Strength

Among the four documents tabulated, the guidance converges on the central obligation, though not on its strength, with each position being recorded in Supplementary Tables S1 and S2 along with its assigned grade. The comparison is illustrative and not exhaustive, as set out in Section 2, and grades are reproduced as each body assigns them rather than harmonised. ACOG recommends at Level A that all women near term with breech presentations be offered an attempt absent contraindications, and RANZCOG makes a Grade A recommendation to the same effect [7,9]. The RCOG holds at Grade B that ECV should be offered from 37 + 0 weeks, backed by an auditable standard targeting 100% of eligible women [8]. The CNGOF recommends on professional consensus only that women be informed of the possibility [51,52], and informing of a possibility and offering a procedure are not equivalent obligations. A fifth professional body, in Canada, recommends the procedure within a document of different remit (Section 8.4) [10].

### 8.2. Divergence Below the Recommendation

Divergence below that level is substantial, on gestational age, tocolysis, neuraxial analgesia and contraindications (Supplementary Tables S1 and S2). The pattern is instructive: where randomised evidence exists, the documents agree; where it does not, they fall back on professional consensus formed within a particular health system. The clearest instance concerns neuraxial analgesia. ACOG states at Level B that it can be considered reasonable with tocolysis, whereas the RCOG states at Grade B that routine use is not recommended while allowing it for a repeat attempt; RANZCOG and the CNGOF do not address it [7,8,9,51,52]. Both colleges are reading the same trials; what separates them is a judgement about proportionality and resource use, the tension identified in Section 7.2 in normative form.

### 8.3. Contraindications and the Eligible Population

The contraindication lists are the mechanism by which guidance shapes the eligible population, and they are heterogeneous in form. RANZCOG provides an explicit taxonomy, the RCOG lists similar exclusions but prefixes them at Grade C with the statement that no general consensus exists on eligibility for, or contraindications to, the procedure, and ACOG and the CNGOF provide no list at all (Supplementary Tables S1 and S2) [7,8,9,51,52]. A systematic review of whether stated contraindications rest on empirical data reached the same conclusion [61]. Across the cohorts in Supplementary Table S3 that report it, the proportion of women with a term breech presentation judged ineligible ranges from 3.9% in a specialist United Kingdom clinic [44] to 22.3% in an Australian population study over a source-defined denominator [46] and 38.5% in a German centre [48]; the Dutch implementation cohort reports 6.6% [14,15]. Case mix, referral selection and the definitions applied differ between these sources, and their effects cannot be separated from the strictness of the criteria themselves (Table 3). No audit of declined or failed procedures identified here reports the proportion excluded. Two guidelines state explicitly that a scarred uterus is not a contraindication [8,51,52].

### 8.4. Guideline Remit and Guideline Silence

A table assembled from comparative reviews rather than primary documents can misrepresent a position that has changed: one widely cited comparison of four national guidelines rests in part on documents since superseded [67]. Four documents are absent from Supplementary Tables S1 and S2. Two could not be assessed: the German national guideline is reported to have expired [65], and no version of the Dutch guideline with verifiable current status was identified. The other two were retrieved and read.

How much a professional body says about external cephalic version depends less on its view of the procedure than on which document it chose to write. Two documents in which the procedure appears once or not at all are set out below, because both are read as national positions when they are closer to editorial decisions.

The stated objective of the Canadian guideline on term breech presentation is to weigh planned vaginal breech birth against planned caesarean and to set selection criteria and delivery technique. It was validated against the RCOG mode-of-delivery guideline rather than the version guideline [10]. Within that remit the document holds that external cephalic version is recommended to reduce the likelihood of caesarean section, at a high quality of evidence. One graded recommendation occurs in a document about how a breech baby is born rather than whether it must be.

The Romanian national guideline sits one step further along. Approved by ministerial order in 2019, it contains no reference to the procedure under any name. It appears in no recommendation and in none of the guideline’s 68 references, while the counselling standard directs the clinician to inform the woman of the two possibilities of birth, vaginal or by caesarean [68]. The document states that its scope does not extend to antenatal care, where the procedure belongs. The omission is a boundary decision and not a rejection of the evidence.

Three claims must be kept apart. That the procedure is absent from a national guideline is a documented fact. That it is therefore not performed does not follow, since no national utilisation data were identified in our search. That women are not offered it is a third claim, and no data bearing on it were identified in our search. What can be said is narrower. Where no document in a health system carries an offer standard, there is nothing to audit and no documented basis on which a clinician can be asked why the procedure was not proposed. Canada and Romania differ in how far along that line they sit, not in kind.

## 9. Counselling and Decision-Making

Counselling determines what proportion of the eligible population reaches an attempt, and is the least standardised element of the pathway. Decision aids improve preference-sensitive decisions across medicine [69], and a breech-specific aid lowered decisional conflict and increased knowledge in a randomised trial [70]. Improved decision quality is a different outcome from increased utilisation, and Section 11 describes what happened when a decision aid was tested against utilisation directly.

Qualitative work identifies a different problem from the one decision aids are designed to solve. In interviews in the north-east of England, most professionals described a personal preference for ECV and counselled directively towards it; several women declined because ECV was not acceptable to them [63]. In an Australian study of women who had undergone an unsuccessful attempt, pain featured prominently in how they subsequently viewed it [71]. Counselling can therefore fail in two directions: insufficiently, leaving a woman unable to make an informed choice, or directively, converting a preference-sensitive decision into a recommendation she is expected to accept. Neither failure is visible in a service that records only whether the procedure was performed.

The content of the conversation is largely settled by the evidence reviewed above. Five elements belong in it:

- A realistic individual probability of success, quoted from parity, engagement and uterine tone rather than as a generic figure [21];
- A probability of vaginal cephalic birth conditional on immediate success of approximately four in five, derived as set out in Section 4 [44];
- The distinction between severe procedure-attributable complications, with estimates of 0.24% in pooled data and 0.45% in a large series with a published event list under strict definitions [43,45], and the broader short-term composites reported at 6% to 10%, which count different events over longer windows and should not be quoted as though they measure the same thing [22,28];
- Pain, which is common and the aspect women most often raise [53,71];
- The alternatives should ECV fail or be declined, namely planned caesarean or, where a service and expertise exist, planned vaginal breech birth [40].

## 10. The Implementation Pathway and Its Measurement

### 10.1. Distinguishing the Steps of the Pathway

**Figure 1:**
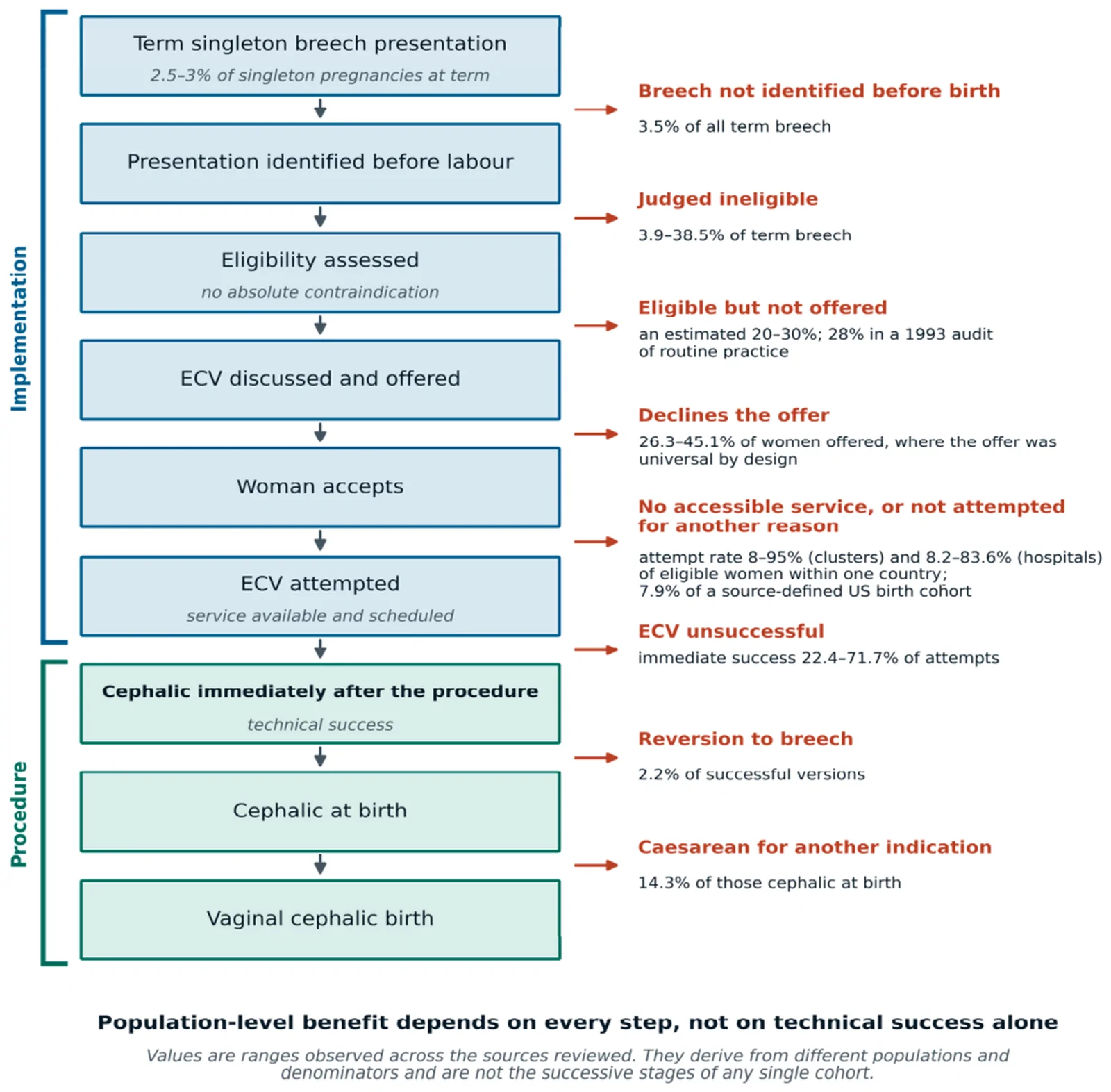
An author-derived conceptual pathway for external cephalic version (ECV), and the points at which eligible women may be lost [7,13,14,15,22,23,25,26,28,43,44,48,53,64,66,73,74]. Original figure.

Section 4, Section 5, Section 6, Section 7, Section 8 and Section 9 established what the procedure delivers when it is performed well. What follows concerns how many women reach it, and the first requirement is to separate the steps at which they do not. To organise these transitions for descriptive and audit purposes we use an author-derived conceptual pathway of nine states, running from a term breech presentation to a vaginal cephalic birth and assembled from the evidence reviewed here rather than taken from a validated model (Figure 1). Four of its transitions are routinely conflated under the single word “uptake”: the eligibility assessment, the offer, itsacceptance and the attempt. This review uses the following definitions throughout. Eligible: women with a term breech presentation in whom no absolute contraindication was identified, over all term breech presentations. Offered: women documented as counselled about and offered an ECV, over eligible women. Accepted: women agreeing to an attempt, over those offered. Attempted: women in whom an attempt was made, over eligible women. Technical success: cephalic presentation immediately after the procedure, over attempts. Downstream benefit: the probability of a vaginal cephalic birth conditional on technical success. These are not interchangeable, and improving one may leave the others untouched.

Three further terms recur and are used in a fixed sense. An *eligibility-assessed denominator* is one constructed by applying eligibility criteria to an identified population of term breech presentations, rather than assembled retrospectively from women already treated or delivering non-cephalic. The *offer rate* is the proportion of eligible women documented as counselled about and offered an ECV, expressed over that denominator. A value reported over any other denominator, or fixed at 100% by study design, is referred to here as an *offer proportion* rather than an offer rate, and its denominator is stated. *Ordinary clinical practice* means care delivered under a service’s routine policy, as distinct from cohorts in which every eligible woman was offered the procedure by study design, where the offer proportion is 100% by construction and describes the protocol rather than the service.

Definitions of success are also inconsistent, which affects comparability more than is generally acknowledged. Most series report presentation immediately after the procedure; a Dutch national programme reported 47% under a composite of success and cephalic presentation at birth [72], and an Australian population study 48.6% based on presentation at delivery [46]. Both are higher on a like-for-like basis than they appear, and neither is comparable with the pooled 58% [43].

In Figure 1, the central column shows the nine successive states between a term breech presentation and a vaginal cephalic birth. The arrows on the right show the attrition that can occur at each transition, with the values reported by the sources reviewed here, shown separately where their denominators differ. Exits may include clinically appropriate contraindication or informed decline, and attrition is not in itself evidence of poor-quality care. The attempt rates shown at the transition to an attempt are cumulative proportions of eligible women rather than losses at that transition, and the 45.1% decline value is expressed over the 102 of 110 women offered who had no other indication for caesarean [23]. The bracket on the left separates the transitions that determine whether a woman reaches the procedure from those that determine what happens once she does. The reversion rate (2.2% of successful versions) and the proportion cephalic at birth (97.3% of successful versions) are reported over slightly different denominators in their source and do not sum to 100%. Values derive from different populations and different denominators and are not the successive stages of any single cohort; the diagram locates where loss occurs and is not a cascade of one population. The nine states are a descriptive scheme assembled by the authors from the evidence reviewed here; neither the sequence nor the states have been externally validated as a measurement instrument.

### 10.2. Magnitude, and What It Can and Cannot Be Compared with

The most informative single figure comes from a cluster randomised implementation trial across 25 Dutch clusters, each a hospital, using their own referring midwifery practices. Of 1613 eligible women, 1169 (72.5%) underwent an attempt, and individual clusters ranged from 8% to 95% [14,15]. A retrospective cohort in the same country reached a similar conclusion by a different route. Across 4770 women in 36 hospitals, an attempt was made in 62.2% of eligible women, with hospital rates from 8.2% to 83.6% [64]. The attempt rate was higher in teaching hospitals. These represent two designs in two periods, with a roughly ten-fold spread each time, in one small country under one national guideline. Data from other health systems rest on different denominators and are descriptive rather than comparable. In New South Wales an attempt was recorded in 13.5% of women classified retrospectively as eligible [46]. In United States birth-certificate data, the rate was 7.9% of 925,082 deliveries classified as ECV-eligible by the source authors [13]. Rates were lower among Black and Hispanic women, unmarried women, women without a college degree and women without private insurance. That United States denominator comprises term singleton births that either had a recorded attempt or were non-cephalic at delivery. The New South Wales population was assembled in the same way, from births with a recorded version or a breech presentation at delivery, with eligibility classified retrospectively from routine records [46]. Neither denominator is interchangeable with the eligibility-assessed denominators of the Dutch series, and neither is treated as such here. Supplementary Table S3 sets out the data by health system.

Whether this loss exceeds what procedural refinement can still add is the question the review was written to ask, and the literature retrieved cannot settle it. The obstacle is not a shortage of studies: the two quantities are of different kinds. A procedural effect is an estimate, a difference in the probability of success between randomised groups, conditional on an attempt having been made. Between-service variation quantifies how attempt rates differ across multiple services per eligible woman, representing purely observational data without predictive modeling. Setting 87 percentage points of dispersion beside the 15 percentage points of immediate success attributable to neuraxial analgesia [31] invites an inference that neither number licences. Rescaling does not repair this: multiplying a procedural effect by an attempt rate would assume the effect constant across services, with services differing only in throughput and the terms independent, and the second assumption is contradicted by the learning-curve data in Section 6.5. Such arithmetic illustrates how the denominators relate; it does not produce an effect estimate.

**Figure 2:**
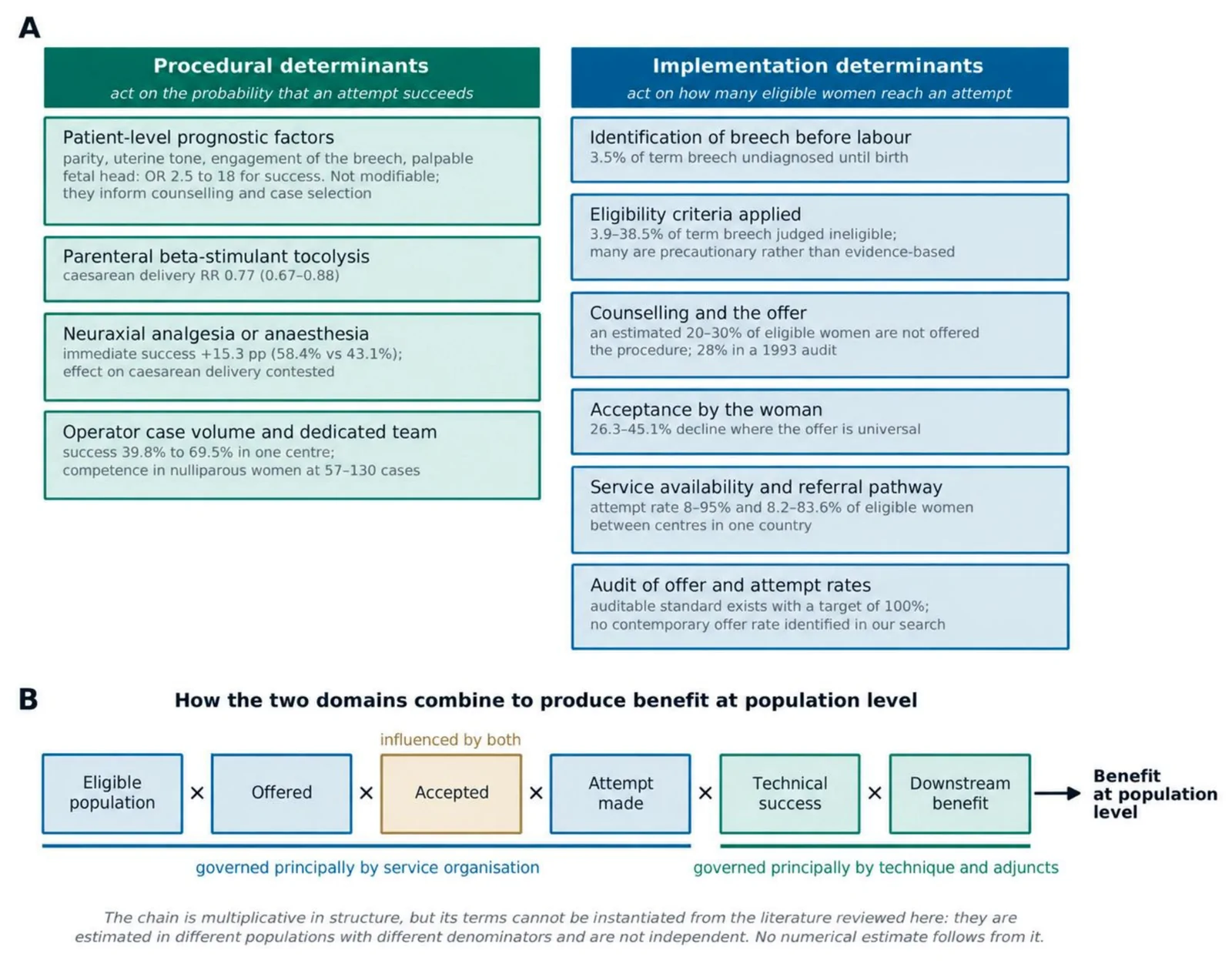
Procedural and implementation determinants of the benefit external cephalic version (ECV) delivers at the population level: (**A**) the determinants operating on each side; (**B**) the chain through which population-level benefit accrues [7,8,14,15,21,23,25,29,30,31,32,44,48,53,61,64,66]. Original figure.

What survives is structural. Every estimate in Table 2 is conditional on an attempt, and the optimisation literature operates inside a denominator whose size is fixed elsewhere, by decisions that the same literature does not measure. Any benefit forgone at a step a woman does not reach cannot be recovered by refining the procedure. Which of the two is the larger source of unrealised benefit is an answerable question, but only for a study measuring both in one population, and we identified no such study in the literature retrieved (Figure 2).

In Figure 2A, procedural determinants act on the probability that a given attempt succeeds; implementation determinants act on how many eligible women reach an attempt. The figure attached to each entry is the effect estimate where one exists and the observed range of the quantity itself where it does not; the two are distinguished in the entry text. Values expressed over different denominators are not combined into a single range. Prognostic factors that are not modifiable, such as parity, appear on the procedural side because they govern the probability of success, but they inform counselling and case selection rather than raising that probability. The 100% target in the audit entry applies only to offering ECV to eligible women [8], not to the proportion of eligible women undergoing an attempt, because informed decline is compatible with appropriate care. The entry for operator case volume and dedicated team rests on one learning-curve analysis [29], which was not replicated in the literature retrieved, and one uncontrolled before-and-after comparison [25], and should not be read as an effect estimate or a validated volume threshold. The 45.1% decline value is expressed over the 102 of 110 women offered who had no other indication for caesarean [23]. In Figure 2B, which shows the chain through which population-level benefit accrues, the domain governing each term is indicated by colour; acceptance, shown in gold, is influenced by both. The fourth term is conditional on acceptance and is not the attempt rate over eligible women used elsewhere in this review. The chain is multiplicative in structure, but its terms cannot be instantiated from the literature reviewed here, because they are estimated in different populations with different denominators and are not statistically independent. No numerical estimate should be read off it.

### 10.3. What the Retrieved Literature Does Not Measure

In the studies retrieved for this review, the offer is rarely measured, and where it is measured the denominator is usually not the eligible population. Of the 19 studies set out in the 18 rows of Supplementary Table S3, four report the offer quantitatively, but none reports an offer rate as defined above. The first is a 1993 audit of routine care, which found that 28% of women known to have a breech presentation and no contraindication were not offered the procedure [66]. Women offered ECV were recorded prospectively, but those not offered were identified by a retrospective review of breech deliveries, so its denominator approximates the eligible population without meeting the definition above. Two are cohorts in which the offer was universal by design, so that the proportion is 100% by construction and carries no information about ordinary clinical practice [23,53]. The fourth reports over a denominator that is not the eligible population as defined here [75]. No contemporary study identified in our search reports the proportion of eligible women offered ECV in ordinary clinical practice over an eligibility-assessed denominator, and none of the population-level studies retrieved report it.

One qualification is owed to the Dutch retrospective cohort. It recorded why an attempt was not made, separating refusal by the woman from contraindication and from the procedure not having been proposed [64]. Among the contemporary studies identified, that is the closest to the measurement argued for here. What it does not report is the offer itself, as a proportion of eligible women, which is the quantity the RCOG standard names [8]. The distinction is narrow and it is the operative one. A service can know why some women went without an attempt while not knowing how many were asked.

**Figure 3:**
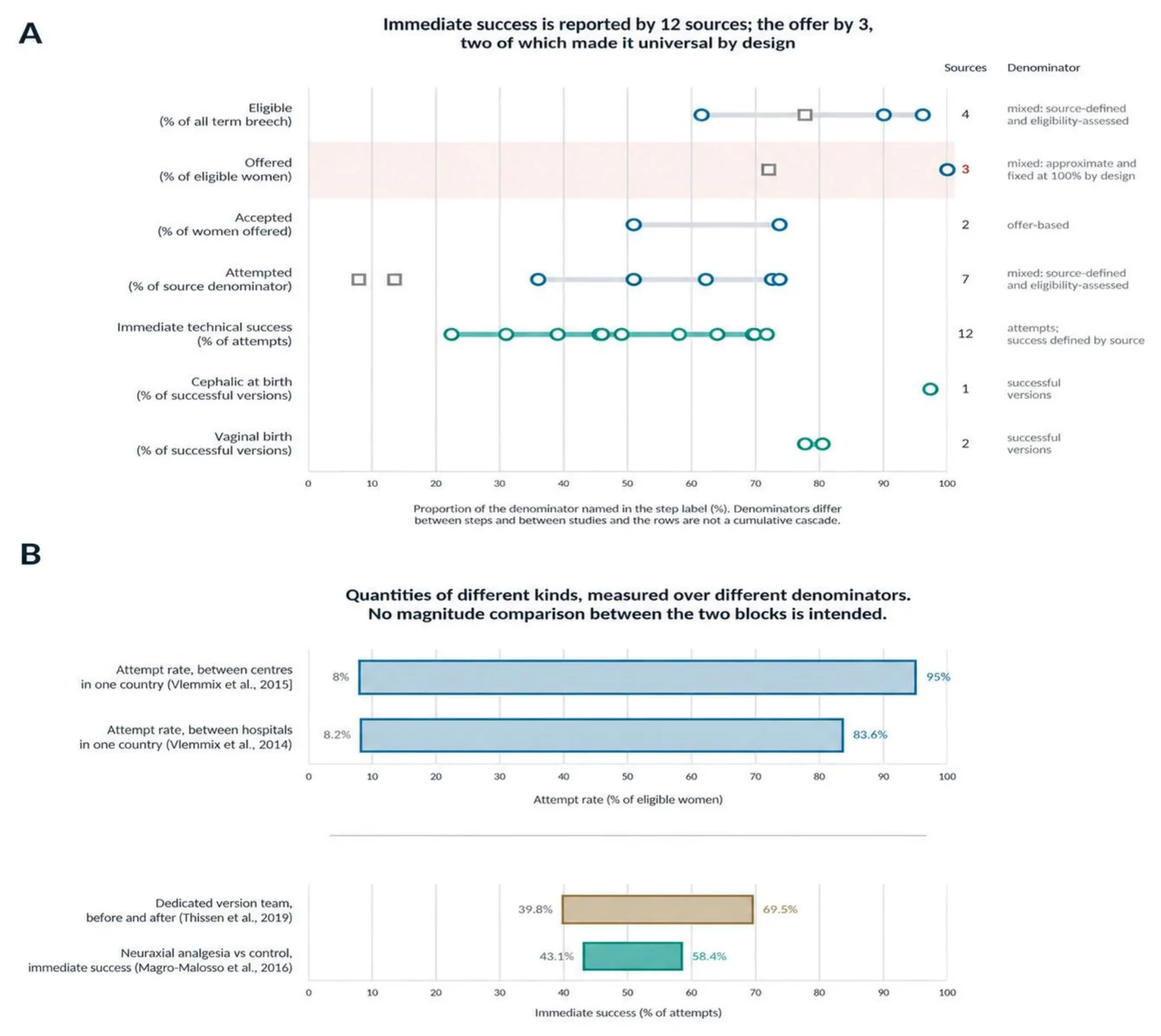
What the retrieved literature measures, and what it does not: (**A**) every value reported for each step of the pathway by the sources reviewed here, plotted as a proportion of the denominator named in the step label; (**B**) two families of quantity, plotted separately because they are not commensurable [14,25,31,64]. Original figure.

The largest population studies substitute an inference for a measurement, deducing selective offering to low-risk women from the lower prevalence of morbid obesity and hypertensive disorders among those who received a version, or from demographic gradients in attempt rates, because the offer itself was not recorded [13,46]. ACOG’s estimate that 20–30% of eligible women are not offered the procedure [7] is the closest thing to a headline figure in the literature retrieved, and it is an estimate and not a measurement. The absence is not for want of a standard: the RCOG lists the proportion of eligible women offered ECV among its auditable topics, with a target of 100% [8]. The 1993 audit, with its partly retrospective denominator, remains the only ordinary-practice source identified that reports a comparable measure, with the two later cohorts having been offered ECV universally by design [23,53]. Technical success is a property of the procedure and the offer a property of the service, and the consequence is that one side of the comparison is systematically invisible in the record retrieved for this review (Figure 3).

The two universal-offer cohorts supply one quantity that ordinary clinical practice cannot: the proportion of women who decline when everyone is asked. Of 167 women under Dutch midwife-led care, all offered an ECV, 123 (73.7%) accepted, so 26.3% declined [53]; in a Polish centre offering the procedure with routine regional anaesthesia, 56 of 110 (50.9%) underwent an attempt and 8 (7.3%) proved to have another indication for caesarean, so 45.1% of the 102 without such an indication declined [23]. Where the offer is guaranteed, therefore, between a quarter and 45% of women still decline. This bounds what counselling alone can achieve and should temper any expectation that universal offering would bring every eligible woman to an attempt.

In Figure 3A, points are individual studies; the first right-hand column gives the number of sources reporting that step, and the second states the kind of denominator each row uses. In the attempt row, grey squares mark the two population values, whose denominators were assembled from births with a recorded attempt or a non-cephalic presentation [13,46]. They are not joined to the eligibility-assessed values. In the offered row, the grey square marks the 1993 value, expressed over a partly retrospective denominator that approximates the eligible population [66], and the circle at 100% marks the two cohorts in which the offer was universal by design [23,53]. In the eligible row, the grey square marks the New South Wales value, expressed over a population assembled from births with a recorded attempt or a non-cephalic presentation, with eligibility classified retrospectively [46]. Rows are not comparable with one another and the steps are deliberately not chained into a cumulative cascade, because no study identified in our search reports the whole pathway: values sharing a horizontal scale do not share a denominator. Two features carry the argument: the offer is reported by three sources against twelve for immediate technical success, and two of those three are cohorts in which the offer was universal by design. Point sources, by step, are as follows: eligible, [15,44,46,48]; offered, [23,53,66]; accepted, [23,53]; attempted, [13,14,23,46,48,53,64]; immediate technical success, [22,23,25,26,28,43,44,48,53,66,73,74]; cephalic at birth, [44]; vaginal birth after a successful version, [22,73]. Values that would require derivation from internally irreconcilable source counts were excluded from the figure. For the Portuguese study the pre-intervention value is plotted, its post-intervention value being 47.9%. The corresponding vaginal-birth value in a third cohort [44] is expressed over women cephalic at birth and is not plotted. A fourth source reports an offer proportion of 65.5% over a 35–37-week breech denominator [75] and is not plotted, since that denominator is not the eligible population defined in Section 10.1. Its success value is excluded from the success row for the same reason, the denominator there being 537 attempts made between 32 and 40 weeks. Values based on composite or at-delivery definitions of success are excluded on the same principle [46,72]. In Figure 3B, the upper block gives observed ranges of attempt rate across services, expressed over eligible women; the lower gives an uncontrolled before-and-after comparison and a pooled randomised estimate, both expressed over attempts. The axes are separate and the blocks should not be read against one another; Section 10.2 sets out why rescaling to a common denominator does not make them comparable.

### 10.4. Patient-Level Determinants

The determinants below are grouped by level, the woman, the clinician and the service, and the grouping is not this review’s invention. It is the structure the Dutch investigators used when they designed the programme from which much of this evidence comes, taken from Fleuren’s determinants-of-innovation framework [76], and their trial was reported against RE-AIM [77]. Naming the framework matters for a reason beyond attribution. Levels are not interchangeable targets. The one randomised implementation trial we identified acted on the first two levels and did not raise utilisation [14], whereas the signals that were positive came from the third. Table 3 sets the three levels out together, with the design and the principal limitation of the evidence for each.

At the level of the woman, the determinants that emerge are informational and experiential rather than dispositional: insufficient information provided at the right moment, apprehension about pain and about harm to the foetus, and a pre-existing preference for planned caesarean [17]. Decline rates can be read reliably only where the denominator is explicit. In the two universal-offer cohorts, where offer and eligibility coincide by design, 26.3% and 45.1% of women offered an attempt declined, with the latter comprising the 102 of 110 women offered an attempt who had no other indication for caesarean [23,53]. A specialist United Kingdom clinic reports the counts a decline proportion would require, but they do not reconcile internally, so no value is derived from them here [44]; the Dutch pre-implementation inventory assumed 20–30% for planning, which is an assumption and not a measurement [78]. The difference between the two measured proportions may reflect counselling context, timing and provider factors, but case mix, service organisation and denominator definition cannot be excluded.

### 10.5. Clinician-Level Determinants

At the level of the clinician, the barriers concern competence and confidence in counselling more than in the manoeuvre. Professionals reported lacking the knowledge to counsel women fully, and specifically an inability to counsel a woman already expressing a preference for planned caesarean [17]; a survey of 364 professionals grouped the determinants of adherence into attitude, professional obligation, outcome expectations, self-efficacy and preconditions [16]. A Polish survey of 461 obstetricians quantifies the result: 78.7% would opt for elective caesarean in a primipara and 73.5% in a multipara, and although 73.8% knew of centres performing ECV, only 16.7% had ever taken part in one [62]. Limited procedural experience may reduce clinician confidence in counselling, and the manner in which counselling is delivered may influence subsequent patient preference; the available observational and qualitative evidence does not establish either relationship causally. That some counsel directively towards ECV [63] points to the same domain from the opposite direction.

### 10.6. System-Level Determinants

At the level of the system, the recurring mechanisms are organisational. The first is the absence of an organised pathway, a hypothesis consistent with the between-cluster spread in Section 10.2 [14]. The second is the concentration of the procedure in few hands, which is associated with higher success but makes access contingent on referral: a German national survey found 98.8% of responding hospitals performed ECV, but respondents were predominantly university and maximum-care hospitals [65]. The third is eligibility criteria whose empirical basis has been questioned (Section 8) [61]. The fourth is the absence of measurement: the investigators who designed the Dutch programme judged the self-reported rates then circulating, of 60–70%, which were probably overestimates precisely because no registration existed [78].

### 10.7. What Is at Stake, and What Is Not Established

One cohort reports the downstream contrast directly: of 1613 eligible Dutch women, 1169 received an attempt and 444 did not, and the caesarean rate was 57% against 87% (RR 0.66; 95% CI 0.62–0.70) [15]. That contrast is observational and confounded by indication, with the bias running upward, since women not offered an ECV or declining it include those already committed to a planned caesarean. The randomised anchor is the Cochrane estimate of RR 0.57 [5], but baseline caesarean risk differed materially between the trial populations and contemporary implementation cohorts, so translating that relative effect onto the 87% observational baseline would not yield a valid absolute estimate. The contrast remains informative about the scale of association and cannot be interpreted causally.

The argument is bounded in two further respects. First, the benefit obtainable by increasing utilisation depends on what would otherwise happen to the woman. Where planned vaginal breech birth is genuinely available from an experienced service, a woman whose foetus remains breech is not committed to caesarean, and the marginal value of an additional ECV is smaller. The German centre in Supplementary Table S3 specialises in vaginal breech birth and reports both a low attempt rate and a low success rate [48]; it is difficult to classify as an implementation failure, and may represent a coherent service design in which ECV plays a smaller part. The benefit at stake is therefore conditional on caesarean being the practical counterfactual, which it is in most high-income settings but not in all.

Second, a retrospective cohort from the Thailand–Myanmar border makes the limits concrete, with the caveat that its exposure window falls partly outside the scope of this review. Among 504 women with a breech presentation at 35–37 weeks, 330 were offered ECV and 174 were not; breech presentation at birth occurred in 50.9% of those offered and 47.7% of those not, with no association after propensity-score adjustment (aOR 1.23; 95% CI 0.82–1.83) [75]. Spontaneous version occurred in 52.3% of women not offered an attempt, a consequence of a cohort beginning at 35 weeks, and success in that service was 50.8%. The result bears on late-preterm rather than term practice, and the offered-against-not-offered comparison is subject to the same selection this review criticises elsewhere. It still makes a point a term-focused implementation argument must accommodate: where spontaneous version is common and procedural success moderate, offering ECV more often does not by itself improve presentation at birth.

## 11. Implementation Strategies: Implications for Services

Any account of what to do next must begin with a negative trial. In the only randomised test of implementation strategies identified in our search, neither a client-directed strategy (leaflet and decision aid) nor a care-provider-directed strategy (a one-day counselling course) improved ECV rates, alone or in combination [14]. The trial did not demonstrate that those interventions work, and suggests that information and counselling of that intensity may be insufficient on their own; it does not establish what the operative constraint is. The 8–95% spread between clusters makes service organisation a plausible alternative candidate, since knowledge deficits are unlikely to be distributed that unevenly between neighbouring centres in one small country, but that remains a hypothesis requiring direct evaluation. A non-randomised evaluation is consistent with that reading. A regional simulation-based training course across ten Portuguese maternity hospitals was followed by ECV services being established in four additional hospitals within two years, yet among the three able to report data, success rates did not change (45.6% versus 47.9%; *p* = 0.797) [73]. Training appears to have created capacity without altering technique, although the design is uncontrolled.

The organisational evidence, though observational, is the most consistent available. A national training programme in the Netherlands delivered 2546 ECV procedures by trained midwives working outside the obstetric theatre, with success in 47% of women under the stricter composite definition described in Section 10.1 and 57% subsequently giving birth vaginally. The reported 2.5% of serious pregnancy outcomes before birth is a composite arising at any point between procedure and delivery, and belongs to the broader tier described in Section 4 [72]. The strongest single-centre evidence is the Dutch hospital described in Section 6.5, where a dedicated team was accompanied by a rise in success from 39.8% to 69.5% between 2013 and 2017, a rise in vaginal delivery after ECV from 43% to 71%, and a fall in caesarean after ECV from 55% to 27% [25]. A Polish centre introducing an ECV service with routine regional anaesthesia reported 39 of 61 attempts (63.9%) as being successful and the unit’s caesarean rate as falling by 2.12 percentage points within a year [23]. The qualitative work underlying the Dutch programme identified, as a facilitator, an unambiguous policy under which ECV is offered in designated centres by specifically trained staff [17].

Read against the learning-curve data in Section 6.5, these findings are consistent with an organisational hypothesis: operator seniority matters less than whether a service is organised so that someone is responsible for the procedure and performs it often enough. Every one of them is either an uncontrolled before-and-after comparison, vulnerable to secular trends and to changes in case selection, or an uncontrolled service evaluation. Service organisation is therefore a plausible organisational determinant that no identified study has evaluated in a randomised comparison, and not a demonstrated cause.

The pattern is not peculiar to this procedure. The WHO recommendations on non-clinical interventions to reduce unnecessary caesarean section, and the Cochrane review underlying them, are organised on the same three levels set out in Section 10.4, Section 10.5 and Section 10.6 [79,80]. External cephalic version is one instance of a problem characterised across obstetrics without being solved.

### Three Process Indicators: A Proposed Reporting Framework

The most tractable intervention may be measurement itself. A 1993 audit approximated the offer rate over a partly retrospective denominator [66], and no contemporary offer rate was identified in the implementation literature retrieved for this review. We propose three process indicators as a pragmatic reporting framework, derived from the gaps identified in this review and intended to be reported alongside the success rate rather than instead of it. The first is the proportion of women with a term breech presentation in whom eligibility was assessed and documented. The second is the proportion of eligible women documented as having been counselled and offered an ECV, which is not a new proposal but an existing audit standard [8]. The third is the proportion of eligible women in whom an ECV was attempted. These are proposed measures and not validated quality indicators. Of the three, only the second carries a benchmark threshold, the target of 100% the RCOG attaches to the offer [8]. We identified no prospective validation of these proposed measures, nor evidence establishing an association with maternal or neonatal outcomes. Each would require evaluation before being treated as a performance measure. A service that reports only its success rate cannot detect losses of the kind described in Section 10, because they occur entirely upstream of the denominator it uses. They are intended for audit: to make visible the point at which eligible women leave the pathway, and to generate hypotheses about why.

## 12. Discussion

### 12.1. Where the Evidence Is Firm and Where It Is Not

The evidence supporting the basic efficacy of term ECV and several procedural adjuncts is comparatively mature, although important technical, safety and implementation uncertainties remain. What is firm can be stated compactly:

- ECV at term reduces non-cephalic presentation at birth and, on balance, caesarean delivery, with certainty for the caesarean outcome being very low [5,6];
- Severe procedure-attributable complications occur in well under 1% under strict definitions, while broader short-term composites reach 6% to 10% and are not comparable with them (Section 4) [22,28,43,45];
- Immediate success is achieved in roughly half to six of ten attempts, and is most consistently associated with parity, uterine tone, engagement of the breech and the palpability of the foetal head [21,24];
- Beta-stimulant tocolysis and neuraxial techniques improve success [30,31,32];
- Success after a previous caesarean is only slightly lower than in an unscarred uterus, within the limits of the data, which are underpowered for uterine rupture [57,58,59];
- Economic modelling places the break-even success rate at approximately one third of attempts [47,49].

Much nonetheless remains open:

- No randomised comparison of the manoeuvres was identified, and standardisation of the technique is itself a research priority [30];
- The limits on attempts rest on evidence that both guidelines specifying them describe as limited [8,9];
- Of 25 prediction models identified, only one has been repeatedly externally validated and judged ready for routine use [11];
- Whether neuraxial techniques reduce caesarean delivery is unresolved across three syntheses [30,31,32];
- Several stated contraindications rest on precaution [61];
- No retrieved study has compared organisational models in a randomised design, and the one randomised implementation trial we identified was negative [14];
- Whether increasing utilisation improves population outcomes has been examined, among the studies identified, only in observational comparisons subject to confounding by indication [15], with one propensity-adjusted analysis in a late-preterm cohort finding no association [75].

The third category is the distinctive one: some quantities are not uncertain so much as unrecorded in the literature retrieved. A 1993 audit approximated the offer rate, identifying women who were not offered retrospectively among breech deliveries [66]. No contemporary study identified in our search reports the offer rate in ordinary clinical practice; the two universal-offer cohorts report an offer proportion that is 100% by construction [23,53]. The reasons a procedure is not proposed, referral failure, service availability and implementation fidelity are inferred or described qualitatively rather than measured [8,13,46], and the denominators over which eligibility, utilisation and success are expressed differ between studies [61,72]. This literature is markedly asymmetrical: the technical evidence is comparatively mature, and the implementation evidence is thin, geographically concentrated, and anchored on the one randomised test we identified, which did not raise utilisation. Whether the upstream loss exceeds the remaining procedural optimisation opportunity cannot be established from the literature retrieved, and no source retrieved describes the measurement infrastructure that would allow it.

Several tensions deserve to be stated openly. The better-tolerated tocolytic is the less effective one [33], and the adjunct with the strongest efficacy evidence is the one whose resource requirements may constrain access [31]. The pooled European and North American determinants were not reproduced in an African series [26], and two of the four highest success rates in Supplementary Table S3 come from the settings with the fewest resources [26,74]. Case mix, selection and outcome definition are each sufficient to generate that pattern, and the published data do not allow the question to be adjudicated.

### 12.2. Limitations

The design is narrative, as set out in Section 2: the search was documented but not exhaustive, screening and extraction were performed by one author, and neither a risk-of-bias assessment nor quantitative pooling was undertaken. Selection bias therefore cannot be excluded. Discordant findings and denominators have been reported wherever the sources permit, and the absence of a contemporary offer rate is an absence in what was retrieved rather than a demonstrated absence in the literature.

The underlying evidence carries further constraints. The meta-analyses anchoring Section 5 were published in 2008 and 2009 [21,24] and the principal safety meta-analysis in 2008 [43], while obstetric practice, case mix and caesarean thresholds have changed since. Definitions of success, of eligibility and of the utilisation denominator differ between studies, and percentages are reproduced as published (Supplementary Table S3). The implementation literature, though drawn from twelve countries and territories, remains weighted towards the Netherlands and one research group, and the non-European evidence comes largely from single centres. Three cohorts cited originate from a single Spanish unit and overlap in the patients they include [22,27,59], and the meta-analyses overlap in their trials, so their estimates are not independent. Finally, the absence of ECV from one national guideline is a statement about that document and not about clinical practice in that country, for which no utilisation data were identified.

### 12.3. Research Priorities

The following research priorities emerge from the gaps identified in this review.

1. Standardised reporting of the pathway: the offer rate alongside the success rate, over an eligibility-assessed denominator, proposed as a core reporting item for future ECV outcome studies [8].
2. A consensus definition of the eligibility-assessed denominator and of technical success, so that rates from different services mean the same thing [61,72].
3. Prospective multicentre audits with an eligibility-assessed denominator, recording eligibility, offer, acceptance, attempt and success separately, and the reason an ECV was not proposed.
4. A study recording offer, attempt and success over explicitly defined denominators in one eligible population in ordinary clinical practice, so that the comparison this review could not make becomes possible.
5. Cluster-randomised trials of service-level interventions, with the offer or attempt rate as primary outcome, powered on between-centre variance; the dedicated-team model is one plausible candidate for prospective evaluation, having the most consistent observational support among the studies identified and not having been randomised in any of them [25,72].
6. A pragmatic trial of selective versus routine neuraxial analgesia, with caesarean delivery as primary outcome and service-capacity consequences measured alongside, with this being the one adjunct whose procedural benefit and resource cost may point in opposite directions [31,32].
7. External validation of the newer prediction models, or a decision to counsel from individual determinants rather than from a composite score [11].
8. Updated pooled syntheses of determinants and complications, as the current ones are approaching two decades old, with a uniform definition of procedure-attributable emergency caesarean [21,24,28,43].
9. Contemporary health-economic evaluation outside the United States, incorporating downstream caesarean avoidance across subsequent pregnancies [42,47,49].
10. Implementation data from health systems where utilisation is low or unmeasured. The evidence we identified from central and eastern Europe is sparse and heterogeneous, comprising a clinician survey, a single-centre service evaluation and a national guideline that does not address the procedure [23,62,68].

These priorities converge on a single study, and it can be specified. It would enrol, across a defined set of maternity services, every woman with a singleton breech presentation confirmed from 36 + 0 weeks. For each woman it would record the four transitions defined in Section 10.1: whether eligibility was assessed, whether the procedure was offered, whether it was accepted and whether it was attempted. Within that same population it would randomise, at the level of the service, an organisational intervention of the kind described in Section 11.

Two outcomes would then be expressed over denominators drawn from one eligible population: the upstream implementation loss, as the attempt rate over eligible women, and procedural performance, as immediate success over attempts. Several studies identified in our search report an attempt rate and success within the same population (Supplementary Table S3), but only the two universal-offer cohorts among them also report the offer, which was fixed by design [23,53]. Powering would rest on the between-service variance in attempt rates, reported at 8% to 95% across Dutch clusters and 8.2% to 83.6% across Dutch hospitals [14,64], which is the largest between-service variation identified in the literature retrieved for this review. Nothing in the design is methodologically novel. What is missing, in the studies identified, is a record of offer, attempt and success over explicitly defined denominators from one eligible population in ordinary clinical practice.

## 13. Conclusions

ECV at term reduces non-cephalic presentation at birth and, on the available randomised evidence, caesarean delivery, although certainty for the caesarean outcome is very low. Severe procedure-attributable complications occur in well under 1% under strict definitions, although broader short-term composites reported by contemporary cohorts are substantially higher and are not comparable with them. Available economic modelling suggests the procedure is cost-effective where success exceeds approximately one third of attempts. Among clinical determinants of procedural success, parity is the most consistently reported, with multiparity associated with more than twice the odds of successful ECV [21]. The largest pooled associations are with findings on clinical examination, including a relaxed uterus, a non-engaged breech and a palpable foetal head, whereas ultrasound determinants show smaller associations [21,24]. What further procedural refinement can add is measured in single-digit to mid-teens percentage points of immediate success, and the extent to which the best-supported adjuncts are implemented in ordinary clinical practice is itself incompletely measured.

The losses that precede the procedure are less well described. Reported attempt rates were 62.2% and 72.5% of eligible women in two Dutch cohorts and 7.9% of a source-defined cohort in United States national data. The denominators differ, and the values are not a direct comparison between health systems. Within one country under one guideline, the spread between centres is roughly ten-fold. No contemporary study identified in our search reports the proportion of eligible women offered the procedure in ordinary clinical practice over an eligibility-assessed denominator, despite an auditable standard with a target of 100%. Procedural effects are estimated over attempts and implementation quantities over eligible women, and we identified no study measuring both within the same population. On the literature retrieved, it is therefore not possible to say which represents the larger source of unrealised benefit, and this review does not claim to have established this. What can be said is narrower: the optimisation literature works inside a denominator it does not measure, and the size of that denominator varies by an order of magnitude between neighbouring services. That asymmetry of measurement, and not any demonstrated ranking of the two losses, is the finding of this review.

Three implications follow for clinical services, each a proposal for local review or evaluation rather than an evidence-based mandate. Precautionary exclusions that lack an empirical or clear pathophysiological basis may be appropriate subjects for local audit, while remaining aligned with applicable national guidance and with individualised clinical assessment. The proportion excluded varies up to tenfold between reported services, although case mix and definitions differ, and every exclusion removes a woman before any conversation occurs. Designated, adequately practised operators are supported by an observational signal, associated with one of the largest observed improvements in success and serving as a plausible contributor to between-centre variation. The supporting designs are before-and-after or uncontrolled, no randomised comparison was identified, and the evidence does not establish that this organisational model improves outcomes. Services auditing their own implementation may report the proportion of eligible women offered ECV alongside technical success. The pathway set out here and the three measures derived from it are proposed audit and reporting tools that require prospective validation, not validated outcome-linked quality indicators.

## Figures and Tables

**Table 1:** Determinants of technical success at external cephalic version. Estimates are reproduced as published and were not pooled for this review; the two meta-analyses used differing definitions of success and are not directly comparable with the single-centre estimates listed alongside them.

Determinant	Direction	Estimate (95% CI)	Design, *n*	Source
Clinical determinants (pooled)				
Relaxed uterus on palpation	Increases success	OR 18 (12–29)	Meta-analysis, 53 studies, *n* = 10,149	[21]
Non-engagement of the breech	Increases success	OR 9.4 (6.3–14)	Meta-analysis, 53 studies, *n* = 10,149	[21]
Palpable foetal head	Increases success	OR 6.3 (4.3–9.2)	Meta-analysis, 53 studies, *n* = 10,149	[21]
Multiparity (parity ≥ 1)	Increases success	OR 2.5 (2.3–2.8)	Meta-analysis, 53 studies, *n* = 10,149	[21]
Maternal weight < 65 kg	Increases success	OR 1.8 (1.2–2.6)	Meta-analysis, 53 studies, *n* = 10,149	[21]
Gestational age at the attempt	No association	Not significant	Meta-analysis, 53 studies, *n* = 10,149	[21]
Fundal height	No association	Not significant	Meta-analysis, 53 studies, *n* = 10,149	[21]
Maternal body mass index	Reduces success	Not pooled ^a^	—	[21,22,23]
Ultrasound determinants (pooled)				
Complete (vs frank) breech	Increases success	OR 2.3 (1.9–2.8) ^b^	Meta-analysis, 37 studies, *n* = 7709	[24]
Posterior placental location	Increases success	OR 1.9 (1.5–2.4)	Meta-analysis, 37 studies, *n* = 7709	[24]
Amniotic fluid index > 10	Increases success	OR 1.8 (1.5–2.1)	Meta-analysis, 37 studies, *n* = 7709	[24]
Determinants from single-centre cohorts				
Transverse or oblique lie	Increases success	OR 3.60 (1.74–7.45)	Cohort, *n* = 990	[22]
Deepest amniotic fluid pocket > 30 mm	Increases success	OR 1.80 (1.04–3.11)	Cohort, *n* = 990	[22]
Nulliparity	Decreases success	OR 0.55 (0.40–0.76)	Cohort, *n* = 990	[22]
Anterior placental location	Decreases success	OR 0.7 (0.48–0.97)	Prospective cohort, *n* = 673	[25]
Absence of pain during the procedure	Increases success	Direction only; reported estimate internally inconsistent in source ^c^	Cross-sectional study, *n* = 152	[26]
Parity; engagement of the presenting part	No association in this setting	Not significant ^c^	Cross-sectional study, *n* = 152	[26]
Sonographic prediction				
Transperineal breech progression angle	Associated but not discriminative	Adjusted OR 0.95 (0.91–0.99); AUC 0.64 (0.53–0.73)	Prospective study, *n* = 117	[27]
Published prediction models	Only one model judged ready for routine use	25 models identified; 13 with no validation, 5 externally validated, 1 externally validated more than once	Systematic review, 25 articles (22 proposing models)	[11]
Service-level determinants				
Operator seniority (years of experience)	No association	Not significant	Retrospective cohort, *n* = 258	[28]
Operator case volume	Increases success ^d^	57 cases for a consistent 50% success rate in nulliparous women, 130 for 70%, 8–10 in multiparous women	Cumulative-sum analysis, *n* = 290	[29]
Dedicated version team	Increases success ^d^	39.8% (2013) to 69.5% (2017), *p* < 0.001; in nulliparous women 23.5% to 58.5%, *p* = 0.002	Prospective cohort; uncontrolled before-and-after comparison, *n* = 673	[25]

AUC, area under the receiver operating characteristic curve; CI, confidence interval; OR, odds ratio. Whether estimates from single-centre cohorts are adjusted or unadjusted is not stated in every source, and they should not be read as carrying weight comparable to the pooled estimates. ^a^ See Section 5: no pooled estimate was identified, and contemporary cohorts report higher body mass index as an independent predictor of failure. ^b^ Directly contradicted by a single prospective cohort of 673 women, in which complete breech was associated with lower success (OR 0.2; 95% CI 0.07–0.90) [25]; see Section 5. ^c^ The point estimates and confidence intervals reported in this source are internally inconsistent as published. Their magnitudes are therefore not reproduced here and are not interpreted quantitatively; only the direction of association, and the absence of a significant association where the source reports one, are carried into the table. No attempt has been made to reconstruct or correct the published values. ^d^ Uncontrolled service-level observations; they do not establish a causal effect or a validated volume threshold.

**Table 2:** Evidence for pharmacological and anaesthetic adjuncts to external cephalic version, grouped by the strength of the evidence supporting them.

Intervention	Comparator	Outcome	Estimate (95% CI)	Absolute Effect	Design, n	Certainty ^a^
Supported by randomised evidence						
Parenteral beta stimulant	Placebo or no treatment	Cephalic presentation in labour	RR 1.68 (1.14–2.48)	—	5 trials, *n* = 459 [30]	Low
Parenteral beta stimulant	Placebo or no treatment	Caesarean delivery	RR 0.77 (0.67–0.88)	—	6 trials, *n* = 742 [30]	Moderate
Parenteral beta stimulant	Placebo or no treatment	Failure to achieve cephalic vaginal birth	RR 0.75 (0.60–0.92)	—	4 trials, *n* = 399 [30]	Moderate
Regional analgesia plus tocolytic	Tocolytic alone	Failed version	RR 0.61 (0.43–0.86)	—	5 trials, *n* = 409 [30]	Moderate
Neuraxial analgesia	No anaesthetic intervention (seven of nine trials) or intravenous analgesia	Successful version	RR 1.44 (1.27–1.64)	58.4% vs. 43.1% (+15.3 pp)	9 RCTs, *n* = 934 [31]	Not graded
Neuraxial anaesthesia	No anaesthesia	Successful version	OR 2.59 (1.88–3.57)	—	Network meta-analysis, 19 trials, *n* = 2296 [32]	Not graded
Neuraxial anaesthesia	Intravenous anaesthesia	Successful version	OR 2.08 (1.36–3.16)	—	Network meta-analysis, *n* = 2296 [32]	Not graded
Neuraxial anaesthesia	Inhalational anaesthesia	Successful version	OR 2.30 (1.33–4.00)	—	Network meta-analysis, *n* = 2296 [32]	Not graded
Effect on caesarean delivery contested						
Neuraxial analgesia	No anaesthetic intervention or intravenous analgesia	Caesarean delivery	RR 0.83 (0.71–0.97)	46.0% vs. 55.3% (−9.3 pp)	9 RCTs, *n* = 934 [31]	Not graded
Regional analgesia plus tocolytic	Tocolytic alone	Caesarean delivery	RR 0.74 (0.40–1.37)	—	3 trials, *n* = 279 [30]	Very low
Any anaesthetic technique	No anaesthesia	Caesarean delivery	No significant reduction	—	Network meta-analysis, *n* = 2296 [32]	Not graded
Head-to-head comparisons between agents						
Atosiban	Beta-agonist	Successful version	RR 0.78 (0.60–0.98)	36.7% vs. 45.3% (−8.6 pp)	4 trials, *n* = 1534 [33]	Not graded
Atosiban	Beta-agonist	Caesarean delivery	RR 1.17 (0.98–1.38)	59.8% vs. 52.6%	4 trials, *n* = 1534 [33]	Not graded
Atosiban	Beta-agonist	Vaginal delivery	RR 0.83 (0.69–1.01)	38.6% vs. 45.0%	4 trials, *n* = 1534 [33]	Not graded
Atosiban	Beta-agonist	Drug-related side effects	RR 0.38 (0.31–0.47)	16.0% vs. 42.9% (−26.9 pp)	4 trials, *n* = 1534 [33]	Not graded
Calcium channel blocker	Beta stimulant	Cephalic presentation at birth	RR 0.62 (0.39–0.98)	—	1 trial, *n* = 90 [30]	Low
Calcium channel blocker	Beta stimulant	Caesarean delivery	RR 1.28 (1.03–1.59)	—	2 trials, *n* = 170 [30]	Low
Nitric oxide donor	Beta stimulant	Cephalic presentation at birth	RR 0.56 (0.29–1.09)	—	1 trial, *n* = 74 [30]	Low
Harms and tolerability						
Neuraxial anaesthesia	No anaesthesia	Maternal hypotension	OR 9.33 (3.14–27.68)	—	Network meta-analysis, *n* = 2296 [32]	Not graded
Neuraxial analgesia	No anaesthetic intervention or intravenous analgesia	Emergency caesarean delivery	RR 0.63 (0.24–1.70)	1.6% vs. 2.5%	9 RCTs, *n* = 934 [31]	Not graded
Neuraxial analgesia	No anaesthetic intervention or intravenous analgesia	Transient foetal bradycardia	RR 1.42 (0.72–2.80)	11.8% vs. 8.3%	9 RCTs, *n* = 934 [31]	Not graded
Regional analgesia plus tocolytic	Tocolytic alone	Foetal bradycardia	RR 1.48 (0.62–3.57)	—	2 trials, *n* = 210 [30]	Low
Intravenous anaesthesia	No anaesthesia	Non-reassuring foetal response	OR 0.36 (0.16–0.82)	—	Network meta-analysis, *n* = 2296 [32]	Not graded
Intravenous anaesthesia	No anaesthesia	Patient satisfaction (VAS)	SMD 1.53 (0.64–2.43)	—	Network meta-analysis, *n* = 2296 [32]	Not graded
Insufficient evidence						
Systemic opioid	Placebo	Failed version	RR 0.77 (0.47–1.26)	—	1 trial, *n* = 60 [30]	Insufficient
Systemic opioid	Regional anaesthesia	Failed version	RR 1.29 (0.93–1.80)	—	1 trial, *n* = 95 [30]	Insufficient
Vibroacoustic stimulation	Placebo	Failed version	RR 0.09 (0.01–0.60) ^b^	—	1 trial, *n* = 23 [30]	Insufficient
Transabdominal amnioinfusion; hypnosis; abdominal lubricants; regional analgesia without tocolysis	Usual care	Successful version	Data insufficient	—	[30]	Insufficient
Methods attempted before version, not adjuncts to it						
Moxibustion plus usual care	Usual care	Non-cephalic presentation at birth	RR 0.87 (0.78–0.99)	—	7 trials, *n* = 1152 [34]	Moderate
Moxibustion	Sham moxibustion	Non-cephalic presentation at birth	RR 0.74 (0.58–0.95)	—	1 trial, *n* = 272 [34]	Moderate
Moxibustion plus usual care	Usual care	Need for external cephalic version	RR 0.62 (0.32–1.21) ^c^	—	4 trials, *n* = 692 [34]	Low
Postural management	Usual care	Non-cephalic presentation at birth	Data insufficient	—	[35]	Insufficient

CI, confidence interval; OR, odds ratio; pp, percentage points; RCT, randomised controlled trial; RR, risk ratio; SMD, standardised mean difference; VAS, visual analogue scale. Risk ratios and odds ratios appear in the same column and are not interchangeable; the measure is stated in every cell. Outcome definitions also differ across sources: “successful version” denotes cephalic presentation immediately after the procedure, whereas cephalic presentation in labour, failure to achieve cephalic vaginal birth and mode of delivery are downstream outcomes. ^a^ Certainty ratings are those assigned by the source review where one was given; “not graded” indicates that the source did not assign a GRADE rating and not that certainty is low. ^b^ The review authors state that this finding cannot be relied upon. ^c^ The source describes the evidence for this outcome as very uncertain while assigning a certainty rating of low; the rating assigned is reproduced here.

**Table 3:** Determinants of how many eligible women reach an ECV attempt, grouped by level. The table is the counterpart of Table 1, which sets out the determinants of whether an attempt succeeds. Values are those reported by the sources named and are not pooled; the designs differ and the final column states what limits the interpretation of each. Denominators differ between entries and are defined in Section 10.1 and Supplementary Table S3.

Level	Determinant	Reported Magnitude	Design of the Evidence	Principal Interpretive Limitation	Source
Woman	Information not provided at a moment when it can be used; apprehension about pain and about harm to the foetus; a pre-existing preference for planned caesarean.	Qualitative themes; no magnitude reported.	Qualitative interview study within a national programme.	Themes are not weighted and cannot be ranked against one another.	[17]
Woman	Declining the offer.	26.3% and 45.1% where the offer was universal by design (45.1% of the 102 of 110 women offered who had no other indication for caesarean); not derived for the specialist clinic, whose source counts do not reconcile.	Retrospective cohort; retrospective analysis; prospective cohort.	The universal-offer values are not generalisable to ordinary clinical practice; the specialist-clinic counts do not reconcile in the source and no proportion is derived from them.	[23,44,53]
Woman	Pain during the procedure.	Significant pain in 34%, of whom 18% also reported fear.	Prospective cohort.	Single setting; pain measured after the fact.	[53]
Clinician	Confidence and competence in counselling rather than in the manoeuvre.	Determinants grouped as attitude, professional obligation, outcome expectations, self-efficacy and preconditions.	Cross-sectional survey of 364 professionals; qualitative study.	Self-reported; response bias not quantified.	[16,17]
Clinician	Preference for planned caesarean, and inexperience of the procedure.	A total of 78.7% would prefer elective caesarean in a primipara and 73.5% in a multipara; 73.8% knew of centres performing ECV but 16.7% had ever taken part in one.	Cross-sectional survey of 461 obstetricians.	Reports stated intention rather than practice; single country.	[62]
Clinician	Directive counselling, in either direction.	Most professionals in one region described counselling directively towards ECV.	Qualitative interview study.	Small and regional; the direction of the effect on acceptance was not measured.	[63]
Service	Absence of an organised referral pathway.	Attempt rate among eligible women 8% to 95% between 25 clusters and 8.2% to 83.6% between 36 hospitals, in one country under one national guideline.	Cluster randomised trial with cohort analysis; retrospective cohort.	Descriptive dispersion, not an effect estimate; no counterfactual attached.	[14,64]
Service	Concentration of the procedure in a small number of centres.	98.8% of responding hospitals reported performing ECV.	National hospital survey, response rate 37.2%.	Respondents skewed towards university and maximum-care hospitals, so the figure describes the centres most likely to offer it.	[65]
Service	Eligibility criteria whose empirical basis has been questioned.	3.9% to 38.5% of women with a term breech presentation judged ineligible in cohorts; 22.3% in one population study over a source-defined denominator.	Cohorts and one population study; one systematic review of the criteria themselves.	Case mix differs between settings and cannot be separated from criterion strictness.	[15,44,46,48,61]
Service	A designated team performing the procedure regularly.	Success rose from 39.8% to 69.5% over four years in one hospital.	Uncontrolled before-and-after comparison.	Vulnerable to secular trends and to changes in case selection; no randomised comparison identified.	[25]
Service	Absence of measurement.	The only ordinary-practice source identified that measures the offer was a 1993 audit whose denominator approximates the eligible population; two later cohorts reported universal offer by design and therefore do not measure ordinary-practice performance. An auditable standard sets a target of 100%.	Audit standard; single 1993 ordinary-practice audit.	Absence in the retrieved literature, not established absence.	[8,66]

Denominators are not uniform across rows and the values are not comparable with one another; each is expressed over the denominator its source used, as recorded in Supplementary Table S3. Nothing in this table is an effect estimate except where the design column states otherwise.

## Data Availability

No new data were created. The quantities derived for this review, and the counts from which they were derived, are set out in Supplementary Table S3 and in Section 10.3; all other data discussed are contained within the cited publications.

## Supplementary Materials

The following supporting information can be downloaded at: https://www.mdpi.com/article/10.3390/healthcare14203452/s1, Table S1: Gestational age, tocolysis, neuraxial analgesia and previous caesarean as recommended in four primary guidance documents; Table S2: Setting, contraindications and the obligation to offer, for the same four documents; Table S3: Reported utilisation of external cephalic version across health systems; Table S4: Principal finding, evidence role and principal interpretive limitation for each study in Table S3.

## Abbreviations

The following abbreviations are used in this manuscript:

Abbreviation	Definition
ACOG	American College of Obstetricians and Gynecologists
aOR	adjusted odds ratio
AUC	area under the receiver operating characteristic curve
CI	confidence interval
CNGOF	Collège National des Gynécologues et Obstétriciens Français
ECV	external cephalic version
GRADE	Grading of Recommendations Assessment, Development and Evaluation
NHMRC	National Health and Medical Research Council (Australia)
NR	not reported
OR	odds ratio
pp	percentage points
QALY	quality-adjusted life-year
RANZCOG	Royal Australian and New Zealand College of Obstetricians and Gynaecologists
RCOG	Royal College of Obstetricians and Gynaecologists
RCT	randomised controlled trial
RE-AIM	Reach, Effectiveness, Adoption, Implementation, Maintenance
RR	risk ratio
SANRA	Scale for the Assessment of Narrative Review Articles
SMD	standardised mean difference
VAS	visual analogue scale
WHO	World Health Organization
