# Supplementary material for: Procedural Optimisation and Unmeasured Implementation in External Cephalic Version at Term: A Narrative Review of a Measurement Asymmetry
Source: Healthcare (Basel). 2026 Oct 15;14(20):3452. doi: 10.3390/healthcare14203452 (PMC13704564; doi:10.3390/healthcare14203452)
Supplement: Supplementary file 1 [file healthcare-14-03452-s001.zip › healthcare-4563772-supplementary.pdf]

Supplementary Materials

Procedural Optimisation and Unmeasured Implementation in External Cephalic Version at Term: A Narrative Review of a Measurement Asymmetry. Tables S1–S4.

Table S1

Gestational age, tocolysis, neuraxial analgesia and previous caesarean as recommended in four primary guidance documents.

Society, document	Document scope and remit	Gestational age and timing	Tocolysis	Neuraxial analgesia	Previous caesarean
ACOG, Practice Bulletin No. 221, 2020 [7]	Practice bulletin devoted to external cephalic version. All seven domains addressed.	Foetal presentation should be assessed and documented from 36+0 weeks to allow for ECV—Level B. Women at ≥37+0 weeks described in the body text as preferred candidates; that statement is ungraded. No conclusion drawn on ECV before 37 weeks.	Evidence supports parenteral tocolysis to improve success—Level B. Terbutaline and parenteral beta stimulants named; data insufficient for calcium channel blockers, and available data discourage nitric oxide donors.	Neuraxial analgesia with tocolytic therapy can be considered a reasonable intervention to increase success—Level B. Data insufficient to evaluate neuraxial analgesia without tocolysis, or to favour spinal over epidural.	Discussed with observational evidence cited (success 50–84%; no uterine rupture reported in the four trials examined). No recommendation and no grade assigned.
RCOG, Green-top Guideline No. 20a, 2nd ed., 2017 [8]	Guideline devoted to the procedure; the companion Green-top 20b covers mode of delivery. Includes auditable standards.	ECV should be offered at term from 37+0 weeks—Grade B. In nulliparous women ECV may be offered from 36+0 weeks—a good practice point. Gestation at term does not affect success and there is no upper limit, although contraindications become commoner.	Tocolysis with betamimetics improves success rates—Grade A. Pragmatic regimens: salbutamol 250 μg intravenously or terbutaline 250 μg subcutaneously. Insufficient data for nifedipine or atosiban against betamimetics; avoid in significant cardiac disease or hypertension; ineffective with beta-blockers.	Routine use of regional analgesia or neuraxial blockade is not recommended, but may be considered for a repeat attempt or for women unable to tolerate ECV without analgesia—Grade B. Supporting text notes reduced failure with regional anaesthesia plus tocolysis (RR 0.61, 95% CI 0.43–0.86) and a less clear effect on caesarean rates.	Women should be informed that ECV after one caesarean delivery appears to carry no greater risk than with an unscarred uterus—Grade C; numbers insufficient to determine the low risk of rupture.
RANZCOG, C-Obs 11, 2021 [9]	Breech-management statement covering antenatal and intrapartum care, with a section on version.	ECV should be offered at term from 37+0 weeks. In nulliparous women a pragmatic approach may be to offer ECV from 36+0 weeks; no clear benefit before 36 weeks; no upper gestational limit, although contraindications may be commoner later. No more than four attempts; maximum 10 minutes overall.	The use of tocolysis improves the success rate of ECV; institutional protocols should define the place of cardiotocography, ultrasound and tocolysis. Not separately graded.	Not addressed in the section on version.	ECV after one caesarean section appears to carry no greater risk than with an unscarred uterus. There were insufficient numbers to determine the low risk of uterine rupture.
CNGOF, 2020 [51,52]	Breech-management guidelines with a chapter dedicated to version (ref. [52]).	Version should be performed from 36 weeks—professional consensus. Supporting text notes that attempts before 37 weeks increase cephalic presentation at birth with a small increase in moderate prematurity.	Intravenous tocolysis is recommended for an attempt at term in order to increase the success rate—Grade B; betamimetics or atosiban.	Not addressed in the section on version.	A scarred uterus is not a contraindication to an attempt at version—professional consensus.

Each cell records the recommendation as its issuing document expresses it, with the grade that document assigns. The four tabulated are those making graded recommendations across the domains compared; the comparison is illustrative and is not an exhaustive survey of national guidance.

ACOG, American College of Obstetricians and Gynecologists; CNGOF, Collège National des Gynécologues et Obstétriciens Français; ECV, external cephalic version; NHMRC, National Health and Medical Research Council; RANZCOG, Royal Australian and New Zealand College of Obstetricians and Gynaecologists; RCOG, Royal College of Obstetricians and Gynaecologists. Grades are reproduced exactly as each document assigns them and are not harmonised: ACOG uses Levels A–C, the RCOG uses Grades A–D with good practice points, RANZCOG uses the NHMRC scheme, and the CNGOF uses evidence grades together with professional agreement. They are not interchangeable, and no grade appears unless the originating document attaches it. Setting, contraindications and the obligation to offer are given in Table S2.

Table S2

Setting, contraindications and the obligation to offer, for the same four documents.

Society, document	Monitoring and setting	Contraindications	Recommendation to offer
ACOG, Practice Bulletin No. 221, 2020 [7]	Foetal well-being and contraction pattern assessed before and after by non-stress test or biophysical profile — Level C. ECV should be attempted only where caesarean services are readily available — Level C. Pre-procedural ultrasound and 30 minutes of post-procedural monitoring described in the protocol text (ungraded). Anti-D for all RhD-negative women whether or not the version succeeds, with stated exceptions (ungraded).	No list provided. The bulletin states that ECV is considered contraindicated where vaginal delivery is not clinically appropriate, that the data are not adequate to establish absolute or relative contraindications, and that these may need to be individualised (ungraded).	All women near term with breech presentations should be offered an ECV attempt if there are no contraindications — Level A, the bulletin's only Level A recommendation. Background text notes that an estimated 20–30% of eligible women are not offered ECV.
RCOG, Green-top Guideline No. 20a, 2nd ed., 2017 [8]	ECV should be performed where facilities for monitoring and surgical delivery are available (good practice point); the standard preoperative preparations for caesarean section are not recommended (good practice point); electronic foetal monitoring after ECV (good practice point); RhD-negative women should undergo testing for fetomaternal haemorrhage and be offered anti-D, minimum 500 IU within 72 h — Grade D. No more than four attempts, maximum 10 minutes overall.	There is no general consensus on eligibility for, or contraindications to, ECV — Grade C. Only placental abruption, severe pre-eclampsia and abnormal foetal Doppler or cardiotocography are supported by evidence; also contraindicated where an absolute indication for caesarean exists, in multiple pregnancy (except after delivery of the first twin), rhesus isoimmunisation, vaginal bleeding within one week, abnormal foetal monitoring, ruptured membranes, or refusal of consent; caution with oligohydramnios or hypertension.	No single recommendation to offer; instead an auditable standard: proportion of women with a breech presentation offered ECV in the absence of contraindications, target 100%. Uptake is best increased by timely identification of the breech presentation and provision of evidence-based information — Grade C.
RANZCOG, C-Obs 11, 2021 [9]	Performed by suitably trained health professionals or by a trainee under direct supervision, in an institution with facilities for emergency caesarean; routine fasting, anaesthetic premedication and intravenous access other than for tocolysis are not recommended as a routine, with individual risk assessment; anti-D within 72 h in RhD-negative women whether or not the ECV is successful — consensus-based recommendation.	Absolute: caesarean required on other grounds; antepartum haemorrhage within 7 days; abnormal cardiotocography; ruptured membranes; rhesus isoimmunisation; multiple pregnancy (except second twin). Relative: small-for-gestational-age foetus with abnormal Dopplers; pre-eclampsia; oligohydramnios; major foetal anomalies; uterine anomalies.	Women with a breech presentation at or near term should be informed about ECV, and ECV should be offered if clinically appropriate — evidence-based recommendation, Grade A (NHMRC), Recommendation 2.
CNGOF, 2020 [51,52]	Foetal heart rate recording before the attempt and for 30 minutes afterwards — professional consensus; no evidence to recommend later or remote monitoring; the attempt should be performed in a facility where emergency caesarean is possible — professional consensus. Routine Kleihauer testing after an attempt is not recommended; RhD prophylaxis to be assured per the college's own alloimmunisation guidance — professional consensus.	No list provided. A single general criterion: an attempt should not be made in situations that justify a planned caesarean for a reason other than breech presentation — professional consensus; a scarred uterus is explicitly not a contraindication.	Women with a foetus in breech presentation should be informed of the possibility of an attempt at version — professional consensus. A clinician without command of the technique should offer referral to another practitioner — professional consensus. The obligation is expressed as one to inform, not to offer.

The documents appear in the same order as in Table S1. The comparison is illustrative and is not an exhaustive survey of national guidance. "Not addressed" is used only where the relevant section of the primary document was checked and the topic is absent. Two further national documents that govern term breech care without making graded recommendations across these domains are discussed in Section 8.4 rather than tabulated, since a row of unfilled cells would misrepresent a document whose remit lies elsewhere. Two documents could not be included at all: the German national guideline is reported to have expired [65], and no version of the Dutch national guideline with verifiable current status was identified.

Table S3

Reported utilisation of external cephalic version across health systems.

Country and setting (year)	Design, n	Eligible, % of term breech	Offered, % of eligible	Accepted, % of those offered	Attempted, % of eligible	Immediate success, % of attempts
United States, national birth-certificate data, 2014–2021 (2026)	Population-based retrospective observational analysis, n = 925,082 source-defined ECV-eligible deliveries [13]	Denominator defined by the authors as ECV-eligible	NR	NR	7.9 k	NR
Netherlands, 25 clusters of hospitals and referring midwifery practices, 2010–2012 (2015, 2016) a	Cluster randomised implementation trial with a companion cohort analysis, n = 1793 breech from 34 weeks, of whom 1613 eligible [14,15]	90.0 (1613/1793); excluded: absolute contraindication 4.0%, other indication for planned caesarean 2.6%, breech undiagnosed until birth 3.5%. Contraindication-based ineligibility is therefore 6.6% b	NR	NR	72.5 (1169/1613); range between clusters 8–95	38.7 i
Netherlands, 36 hospitals, 2008–2009 (2014)	Retrospective cohort of all singleton breech deliveries from 36 weeks, n = 4770 [64]	Eligibility assessed by chart review; proportion of term breech not reported	NR	NR	62.2 (2443 of eligible women); range between hospitals 8.2–83.6	NR
Netherlands, midwife-led primary care (2010)	Prospective cohort, n = 167 women with confirmed breech from 33 weeks [53]	NR	100 c	73.7 (123/167; 95% CI 65.5–80.5)	73.7	39
Netherlands, national midwife training programme, 2008–2011 (2016)	Prospective multicentre observational study, n = 2546 versions [72]	NR	NR	NR	NR	47 d (34% in nulliparous, 66% in multiparous women)
Australia, New South Wales, whole state, 2002–2012	Population-based record linkage, n = 32,321 singleton breech pregnancies [46]	77.7 k (22.3% ineligible)	NR	NR	13.5 k	48.6 f
United Kingdom, single teaching hospital, 16-month audit (1993)	Prospective database with retrospective review of all breech deliveries [66]	NR	72 l (39 of 137 women without a contraindication were not offered an attempt: 28%)	NR	NR	46
United Kingdom, Oxford specialist clinic, 18 years (2019)	Retrospective cohort of prospectively collected data, n = 3008 women with confirmed breech [44]	96.1 (117 of 3008, 3.9%, ineligible)	NR	Not calculated; source counts do not reconcile g	Not calculated; source counts do not reconcile g	49.0 (95% CI 47.0–50.9); 40 in nulliparous, 64 in other women
Poland, national survey of obstetricians (2025)	Cross-sectional online survey, 461 respondents [62]	NR	NR	NR	NR	NR
Poland, single centre, Katowice, 2023–2024 (2025)	Retrospective analysis of a newly introduced service, n = 110 women meeting criteria [23]	NR	100 c	50.9 h (56/110; 46 declined, 8 had another indication for caesarean)	50.9	63.9 (39 of 61 attempts) h
Germany, national hospital survey (2020)	Anonymised online survey of 234 hospitals, response rate 37.2% [65]	NR	NR	NR	NR	NR
Germany, tertiary centre specialising in vaginal breech birth, 2013–2017 (2021)	Prospective cohort with retrospective data acquisition, n = 1598 women presenting for breech birth planning [48]	61.5	NR	NR	~36† e	22.4
Portugal, 10 public maternity hospitals (2024)	Before-and-after intervention study following a regional simulation-based training course [73]	NR	NR	NR	NR	45.6 before vs 47.9 after (p = 0.797)

Country and setting (year)	Design, n	Eligible, % of term breech	Offered, % of eligible	Accepted, % of those offered	Attempted, % of eligible	Immediate success, % of attempts
Canada, tertiary centre (2024)	Retrospective chart review, n = 258 [28]	NR	NR	NR	NR	31
Spain, tertiary centre, 2014–2024 (2026)	Retrospective cohort study under a standardised protocol with tocolysis and sedation or spinal anaesthesia, n = 990 [22]	NR	NR	NR	NR	69.8
Ethiopia, tertiary centre, Addis Ababa (2023)	Cross-sectional study, n = 152 [26]	NR	NR	NR	NR	71.7
India, secondary hospital, Vellore (2020)	Retrospective review, 2012–2020, n = 200 [74]	NR	NR	NR	NR	64.0
Thailand–Myanmar border, migrant and refugee clinics, 2008–2018 (2026)	Retrospective cohort with propensity-score analysis, n = 504 women with breech at 35–37 weeks [75]	NR	65.5 j (330/504)	NR	NR	50.8 j (273/537 attempts, 32–40 weeks)

Each step of the author-derived pathway (Section 10.1) is shown separately where the source reports it.

CI, confidence interval; ECV, external cephalic version; NR, not reported. NR means that the study did not report the quantity, not that the quantity was zero or inapplicable. Column denominators are as defined in Section 10.1; where a source used a different denominator the cell is footnoted. Unless explicitly identified in the table with a dagger (†) and in footnote e as author-derived, percentages are reproduced as published and were not recalculated to a common denominator, because the underlying counts are not available for every study. The pattern of NR entries is itself a finding of this review: immediate success is reported almost universally, whereas no study tabulated reports an offer rate as defined in Section 10.1. Table S4 gives the principal finding, the design and the interpretive limitation for each row.

a The cluster randomised trial and the companion cohort analysis report the same 1169 attempts among the same 1613 eligible women and are presented here as one entry to avoid double counting. The two reports differ on the lower gestational bound of the source population, 32 weeks in [14] and 34 weeks in [15]; the value is reproduced as given in [15]. b The 10.0% excluded includes 3.5% of pregnancies in which the breech was undiagnosed until birth, which is not an eligibility judgement; 6.6% is the proportion excluded on contraindication or on an existing indication for caesarean, and is the figure comparable with the other entries in this column. c The offer was universal by design, so this value is 100% by construction and carries no information about ordinary clinical practice. d Success defined in this study as cephalic presentation immediately after the procedure and at birth, a stricter composite than the immediate-success definition used elsewhere in this review; the rate is therefore not comparable with the other values in this column. e Calculated for this review and not reported as such by the source: the German figure divides 353 attempts by 61.5% of 1598 women. f Based on foetal presentation at delivery and not immediately after the procedure. g The source's own counts do not reconcile: 3008 − 117 ineligible − 297 declining leaves 2594, against 2614 women reported as undergoing an ECV. Neither an acceptance nor an attempt proportion is therefore entered for this cohort, and no decline proportion is derived from these counts in Section 10.4. The counts are reproduced as published and no attempt has been made to reconstruct or correct them. Values reported directly by the source and independent of this discrepancy, including the proportion judged ineligible and the immediate success rate, are retained. h 56 women underwent 61 attempts; the success denominator is attempts and not women. Over the 102 women without another indication for caesarean, 45.1% declined (calculated for this review; the source reports 46 of all 110 women offered, 41.8%); the accepted value is expressed over all 110 women offered, so the two proportions are not complementary. i Reported by [15] as a mean success rate across the cohort rather than as a per-attempt proportion over a stated denominator, and is not plotted in Figure 3A for that reason. j The denominator for the offer is women with a breech presentation at 35–37 weeks and not eligible women as defined here; the success denominator is 537 attempts made between 32 and 40 weeks. Both fall partly outside the term scope of this review, and neither is plotted in Figure 3. k Denominator assembled by the source authors from births with a recorded ECV attempt or a non-cephalic presentation at delivery, with eligibility classified retrospectively from routine records. It is not an eligibility-assessed denominator as defined in Section 10.1, and these values are not comparable with those of the Dutch cohorts. The New South Wales attempt proportion is reported by the source [46]. l Women offered ECV were recorded in a prospective database; women not offered were identified by a retrospective review of breech deliveries. The denominator therefore approximates the eligible population without meeting the definition in Section 10.1.

Table S4

Principal finding, evidence role and principal interpretive limitation for each study in Table S3.

Country and setting (year)	Principal finding	Evidence role and principal limitation
United States, national birth-certificate data, 2014–2021 (2026)	Attempt rates lower among Black and Hispanic women, unmarried women, women without a college degree and women without private insurance; the authors conclude that ECV is underutilised and not uniformly offered, inferring selective offering from those gradients	Population-based retrospective observational analysis of routinely collected birth-certificate data. Denominator defined by the source authors; the offer was not recorded and selective offering is an inference.
Netherlands, 25 clusters of hospitals and referring midwifery practices, 2010–2012 (2015, 2016) a	Neither a client strategy (leaflet and decision aid) nor a care-provider strategy (one-day counselling course) improved the rate. Caesarean rate 57% in women who received an attempt versus 87% in those who did not (RR 0.66, 95% CI 0.62–0.70), an observational and confounded comparison	Cluster randomised trial with companion cohort analysis. The randomised comparison concerns the implementation strategies; the utilisation and caesarean figures are observational and confounded by indication.
Netherlands, 36 hospitals, 2008–2009 (2014)	The attempt rate was higher in teaching hospitals. The study documented reasons for not performing an attempt, and is the closest any contemporary source identified comes to measuring the offer, though it does not report the offer as a proportion of eligible women (Section 10.3)	Retrospective multicentre cohort. Chart-based, no comparator, hospital characteristics analysed observationally.
Netherlands, midwife-led primary care (2010)	One of the two cohorts identified in which the offer was universal by design, so that the loss is attributable to acceptance and not to offering; significant pain reported by 34% of women	Small prospective single-setting cohort. Universal offer by design, so the acceptance figure does not describe ordinary clinical practice.
Netherlands, national midwife training programme, 2008–2011 (2016)	ECV performed safely by trained midwives outside the obstetric theatre; 57% of women who underwent an ECV gave birth vaginally	Prospective multicentre observational study of a training programme. No comparator; success defined by a stricter composite than elsewhere in this review.
Australia, New South Wales, whole state, 2002–2012	Caesarean followed 25.9% of successful and 95.6% of unsuccessful attempts; the authors infer selective offering to low-risk women from the lower prevalence of morbid obesity and hypertensive disorders among women who received an ECV	Population-based record-linkage study. The population was assembled from births with a recorded ECV or a breech presentation at delivery, with eligibility classified retrospectively. Strong for utilisation, no offer denominator; selective offering is an inference from case mix.
United Kingdom, single teaching hospital, 16-month audit (1993)	The only ordinary-practice source identified that measures the offer: 28% of women known to have a breech presentation and no contraindication were not offered ECV; no later measurement of this kind was identified in our search	Single-centre audit, 1993, combining a prospective database of women offered ECV with a retrospective review of breech deliveries. Measures the offer in ordinary clinical practice, unlike the two later cohorts in which the offer was universal by design, but over a denominator that only approximates the eligible population; small and historical.
United Kingdom, Oxford specialist clinic, 18 years (2019)	97.3% of successful versions were cephalic at birth and 85.7% of those women delivered vaginally; the source also reports 297 women declining, but the counts a reliable decline or attempt proportion would require do not reconcile; women with a previous caesarean were not referred to the clinic	Large single-centre retrospective cohort of a specialist clinic, highly selected. Source counts do not reconcile internally, so no acceptance or attempt proportion is derived from them.
Poland, national survey of obstetricians (2025)	For a term non-cephalic presentation, 78.7% would prefer elective caesarean in a primipara and 73.5% in a multipara; ECV would be chosen by 21.3% and 23.6%. 73.8% knew of centres performing ECV, but only 16.7% had ever taken part in one	Cross-sectional clinician survey. Reports stated intention, not practice; response rate and self-selection unquantified.
Poland, single centre, Katowice, 2023–2024 (2025)	The unit's overall caesarean rate fell by 2.12 percentage points within the first year; an uncontrolled feasibility signal and not an effect estimate	Uncontrolled single-centre retrospective before-and-after service evaluation. The caesarean figure is a feasibility signal, not an effect estimate.
Germany, national hospital survey (2020)	98.8% of responding hospitals perform ECV, but respondents were predominantly university (26.4%) and maximum-care (35.6%) hospitals, so the figure describes the centres most likely to offer it. The authors note that German practice rests on an expired national guideline	Cross-sectional hospital survey, response rate 37.2%. Respondents skewed towards tertiary centres, so the figure describes those most likely to offer the procedure.
Germany, tertiary centre specialising in vaginal breech birth, 2013–2017 (2021)	Both the denominator and the low success rate reflect selection in a centre where planned vaginal breech birth is routinely available; ECV performed at 38 weeks	Single-centre prospective cohort in a service specialising in vaginal breech birth; both denominator and success rate reflect that selection.
Portugal, 10 public maternity hospitals (2024)	ECV services were established in four additional hospitals within two years, but success rates did not change in the three hospitals able to report data; 77.7% of women with a successful ECV delivered vaginally	Multicentre before-and-after evaluation of a training intervention. Only three of ten hospitals could report outcome data.
Canada, tertiary centre (2024)	Emergency caesarean 10% and neonatal unit admission 8%, both higher than the authors anticipated. The source defines the outcome as emergent caesarean delivery due to ECV, so it is procedure-attributable by the authors' own definition, but over an observation window and an event set that differ from those of the pooled 0.35% (Section 4)	Single-centre retrospective chart review. Complication outcomes defined by the source as procedure-attributable.
Spain, tertiary centre, 2014–2024 (2026)	80.5% of women with a successful ECV delivered vaginally. Complications occurred in 101/990 (10.2%), including 66/990 (6.7%) emergent caesarean sections, a broader composite than the strictly defined severe events pooled in [43] and not comparable with them (Section 4)	Large single-centre cohort under a standardised protocol, designated a retrospective cohort study by its authors, with data collection partly retrospective and partly prospective (2014–2020 and 2020–2024, respectively). No comparator.
Ethiopia, tertiary centre, Addis Ababa (2023)	Parity and engagement of the presenting part were not associated with success in this setting	Single-centre cross-sectional study. Small; determinant analysis underpowered and reported estimates are internally inconsistent.

Country and setting (year)	Principal finding	Evidence role and principal limitation
India, secondary hospital, Vellore (2020)	Reported as feasible in a limited-resource secondary setting; barriers to offering not assessed	Single-centre retrospective review in a limited-resource setting. No offer denominator; barriers not assessed.
Thailand–Myanmar border, migrant and refugee clinics, 2008–2018 (2026)	Breech at birth 50.9% in women offered ECV versus 47.7% in those not offered (adjusted OR 1.23, 95% CI 0.82–1.83); spontaneous version occurred in 52.3% of women not offered an attempt	Retrospective cohort with propensity-score adjustment. Contextual only: the exposure window falls partly outside the term scope of this review.

Rows appear in the same order as in Table S3. The designs differ and are not to be read as carrying equivalent weight; this column is provided so that the reader can distinguish randomised evidence, population cohorts, retrospective service audits, before-and-after evaluations, surveys and qualitative work without a formal risk-of-bias assessment, which was not undertaken (Section 2).
